# Taxonomic and functional shifts in the beech rhizosphere microbiome across a natural soil toposequence

**DOI:** 10.1038/s41598-017-07639-1

**Published:** 2017-08-29

**Authors:** Y. Colin, O. Nicolitch, J. D. Van Nostrand, J. Z. Zhou, M.-P. Turpault, S. Uroz

**Affiliations:** 1 INRA, Université de Lorraine, UMR 1136 “Interactions Arbres Micro-organismes”, Centre INRA de Nancy, 54280 Champenoux, France; 2 INRA UR 1138 “Biogéochimie des Ecosystèmes Forestiers”, Centre INRA de Nancy, 54280 Champenoux, France; 3 Institute for Environmental Genomics, and Department of Microbiology and Plant Biology, University of Oklahoma, Norman, OK 73072 USA; 4 Earth Sciences Division, Lawrence Berkeley National Laboratory, Berkeley, CA 94720 USA; 5 State Key Joint Laboratory of Environment Simulation and Pollution Control, School of Environment, Tsinghua University, Beijing, 100084 China

## Abstract

It has been rarely questioned as to whether the enrichment of specific bacterial taxa found in the rhizosphere of a given plant species changes with different soil types under field conditions and under similar climatic conditions. Understanding tree microbiome interactions is essential because, in contrast to annual plants, tree species require decades to grow and strongly depend on the nutritive resources of the soil. In this context, we tested using a natural toposequence the hypothesis that beech trees select specific taxa and functions in their rhizosphere based on the soil conditions and their nutritive requirements. Our 16S rRNA gene pyrosequencing analyses revealed that the soil type determines the taxa colonizing the beech rhizosphere. A rhizosphere effect was observed in each soil type, but a stronger effect was observed in the nutrient-poor soils. Although the communities varied significantly across the toposequence, we identified a core beech rhizosphere microbiome. Functionally, GeoChip analyses showed a functional redundancy across the toposequence, with genes related to nutrient cycling and to the bacterial immune system being significantly enriched in the rhizosphere. Altogether, the data suggest that, regardless of the soil conditions, trees enrich variable bacterial communities to maintain the functions necessary for their nutrition.

## Introduction

Plants are known for their ability to colonize a wide range of terrestrial environments and to adapt to various climatic or edaphic constraints^1^. As an example, the distribution of deciduous and coniferous trees in forest ecosystems is determined by local environmental factors such as climate, water availability and soil type^2, 3^. In boreal regions, forests are dominated by coniferous tree species adapted to low temperatures, such as *Picea abies*. In contrast, temperate regions are dominated by *Fagus sylvatica* L., which presents a broader area of distribution despite having a high sensitivity to drought and high temperatures^4–6^. The broad distribution of beech in soils developed on calcareous to acidic mineral parental materials is currently explained by its high tolerance to various soil parameters such as pH, nutrients availability, water content compared to other tree species^6^. Notably, several studies have highlighted that beech trees harbour variable densities of fine root biomass (roots devoted to nutrient access) in the topsoil depending on the soil acidity and/or nutrient availability^6–8^. Such adaptation of beech trees to acidic conditions, which are characterized by low nutrient availability, suggests a strong investment by this tree species in soil exploration and nutrient access.

The adaptation of beech trees to low nutrient soils may be partly due to its ability to produce root exudates and to select within its root vicinity microorganisms capable of accessing nutrients^9^. Indeed, plants are known to produce a wide range of compounds, including protons, organic acids, amino acids, carbohydrates, phytosiderophores and signal molecules^10–12^. Through these compounds, the production of which is regulated by plant age, root maturity and nutrient availability, plants modify the soil physico-chemical characteristics to fit their nutritional requirements^10, 13–16^. In this sense, Augusto *et al*.^17^ revealed that trees impact soil chemistry, showing that coniferous trees strongly increase nutrient availability compared to deciduous species such as beech or oak through the acidification of the soil. Comparisons of the bulk soil and rhizosphere compartments below beech, Douglas fir or Norway spruce revealed an increase in the nutrient availability in the rhizosphere compared to the bulk soil, suggesting that active processes occur in the rhizosphere to access nutritive elements entrapped in organic matter and soil minerals^18, 19^. In addition to acidification process, the rhizosphere provides a carbon-rich environment that is favourable to bacterial communities^11, 16, 20–25^. Indeed, the enrichment of specific bacterial communities in the rhizosphere has been well-established for a wide range of perennial and non-perennial plants^26–33^. Notably, the enriched communities in the rhizosphere harbour functional traits capable of improving nutrient cycling, plant nutrition or protection against pathogens, suggesting plants select effective bacterial partners in their rhizosphere^34, 35^. Such selection has been evidenced in acidic and nutrient-poor forest soils, showing the enrichment of effective mineral-weathering bacteria in the rhizosphere of various tree species, including beech, oak and Norway spruce^9, 36, 37^. This selective process was also reported for non-perennial plants and in other ecosystems^38–41^. These studies highlighted that the bacterial communities occurring in the soil reservoir vary based on the soil type and its physico-chemical properties, and especially the pH^38, 39, 41–44^. However, it remains extremely difficult to disentangle whether the observed effect is directly related to pH or indirectly affected by variation in other co-varying edaphic parameters^41^. Indeed, soil pH modulates other edaphic parameters such as nutrient availability, aluminium availability, organic carbon and phosphorus^45^, which may in turn influence soil bacterial communities.

Altogether, these studies clearly demonstrate that soil edaphic parameters and the tree rhizosphere each strongly determine the diversity, structure and functioning of soil bacterial communities^33, 43^. However, the extent to which both associated factors contribute to shaping forest soil microbiomes is not fully understood and is likely to vary depending on the edaphic conditions and tree physiology. Understanding tree microbiome interactions is essential as, in contrast to annual plants, trees need decades to grow and strongly depend on the nutritive resources in the soil. Moreover, forests are rarely amended, making the accessibility and recycling of nutrients key processes for the long-lasting development of trees. In this context, our objectives in this study were (i) to determine the structure and functional potential of bacterial communities in the rhizosphere of a common European tree species (*Fagus sylvatica*), (ii) to assess how the structure and function of these bacterial communities change in relation to soil properties and (iii) to test if a core beech rhizosphere microbiome exists regardless of the soil conditions. The study was carried out at the Montiers Long-Term Observatory (LTO). This site is characterized by a natural soil toposequence ranging from low nutrient availability and acidic pH to high nutrient availability and neutral pH, colonized by the same land cover, which is dominated by beech (*Fagus sylvatica L*.) trees. Due to forestry practices, the trees are of similar age with an average age of 55 years. Such a toposequence is interesting because it presents contrasting soil conditions that reflect the range of soils on which forest environments have developed in Europe on a broader scale. We hypothesized that in soils presenting different physico-chemical properties and different bacterial communities, *F*. *sylvatica* L. trees select specific bacterial communities in their rhizosphere based on the nutrient availability and their nutritive requirements. To assess the community structure and richness, we performed 16S rRNA gene amplicon sequencing on DNA isolated from the rhizosphere and bulk soil samples. The same soil samples were also used to assess the functional potential and functional richness of bacterial communities using GeoChip 5.0 microarrays and Biolog Ecoplates and to perform soil chemical analyses.

## Results

### Soil characteristics and Biolog analysis

#### Edaphic parameters

The Calcaric plots were characterized by neutral pH values and the highest contents of organic matter (OM), carbon (C), nitrogen (N), limestone and exchangeable nutritive cations (Table S1). In contrast, pH values of 5 and 4.6 characterized the Eutric and Hyperdystric plots, respectively. The cation-exchange capacity (CEC) strongly decreased in both these plots compared to the Calcaric plots. The amount of exchangeable nutritive cations such as calcium (Ca), magnesium (Mg), potassium (K) and sodium (Na) were also significantly reduced compared to the Calcaric plots (*P* < 0.001; ANOVA) (Table S1). Moreover, significantly more aluminium (Al) (*P* < 0.001; ANOVA) and manganese (Mn) (*P* < 0.05; ANOVA) were measured in acidic soils (Hyperdystric and Eutric).

#### Substrate utilization assays

The analysis of the metabolic potentials revealed a decreasing metabolic diversity gradient from Calcaric to Hyperdystric soils (P < 0.05) based on the Shannon-Weaver index (H′) calculation. In Calcaric soils, no difference in metabolic diversity was observed between the bulk soil and rhizosphere samples (Figure S1), while the rhizosphere-associated microbial communities of the two other soil types displayed a lower metabolic diversity than those of the bulk soil. The microbial communities inhabiting the Calcaric soil showed significantly higher degradation potentials for glycogen, 2-hydroxybenzoic acid, phenylalanine and putrescine than those of the two other soil types (*P* < 0.05). In the Hyperdystric soil, carbon substrates such as asparagine, Tween 40, Tween 80 and pyruvic acid methyl ester were significantly more metabolized in the rhizosphere compared to the surrounding soil (*P* < 0.05). Lastly, the bulk soil samples from the Hyperdystric soil metabolized significantly more glucose-1-P and D-galacturonic acid than their corresponding rhizosphere samples (*P* < 0.05).

### Bacterial diversity and richness

#### 16S rRNA gene libraries

**Figure 1:**
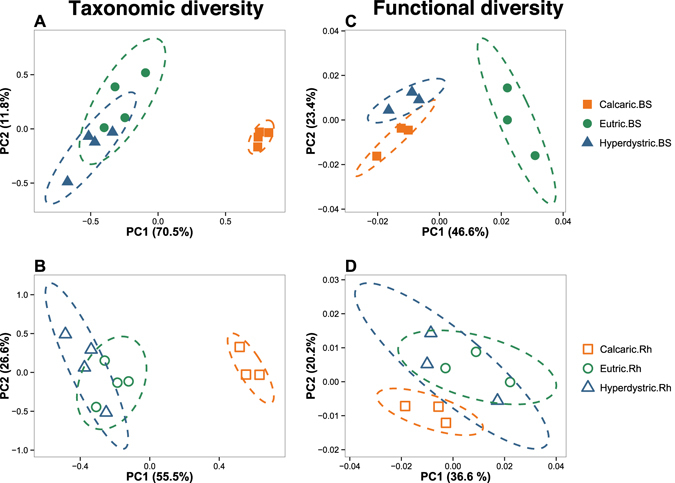
Shift in taxonomic and functional diversity of bulk soil and rhizosphere-associated bacterial communities along the soil toposequence of Montiers. Multivariate analyses were performed at the phylum level for the 16S rRNA gene libraries (**A** and **B**) and at the subcategory level for the GeoChip microarray (**C** and **D**). For both approaches, multivariate analyses were conducted separately on bulk soil (BS) and rhizosphere (Rh) samples. The origins of samples are indicated as follows: filled orange squares, bulk soil samples from Calcaric; open orange squares, rhizosphere samples from Calcaric; filled green circles, bulk soil samples from Eutric, open green circles, rhizosphere samples from Eutric; filled blue triangles, bulk soil samples from Hyperdystric; open blue triangles, rhizosphere samples from Hyperdystric. Ellipses correspond to 95% confidence intervals about the mean.

**Figure 2:**
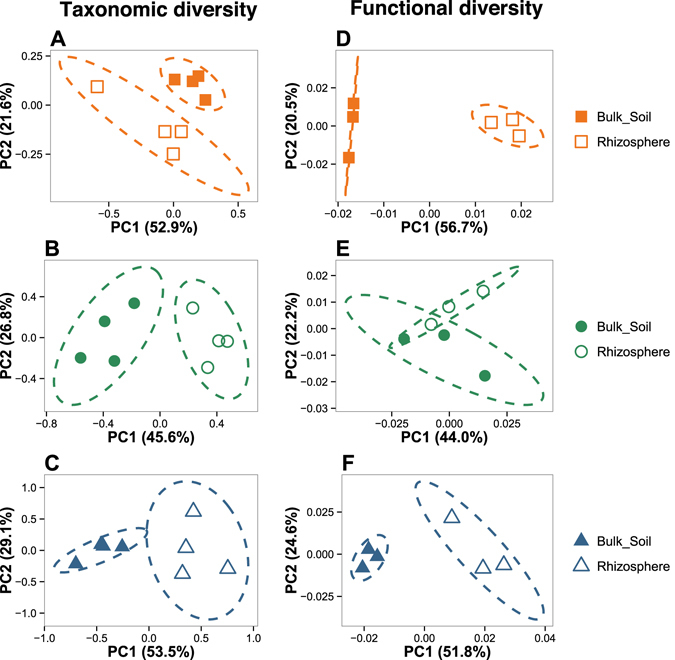
Shift in taxonomic and functional bacterial diversity between bulk soil and rhizosphere compartments in each soil type. Multivariate analyses were performed at the phylum level for the 16S rRNA gene libraries (**A**,**B** and **C**) and at the functional subcategory level for the GeoChip dataset (**D**,**E** and **F**). For both approaches, multivariate analyses were conducted separately on each soil types to determine a potential rhizosphere effect. The origins of samples are indicated as follows: filled orange squares, bulk soil samples from Calcaric; open orange squares, rhizosphere samples from Calcaric; filled green circles, bulk soil samples from Eutric, open green circles, rhizosphere samples from Eutric; filled blue triangles, bulk soil samples from Hyperdystric; open blue triangles, rhizosphere samples from Hyperdystric. Ellipses correspond to 95% confidence intervals about the mean.

The analysis of the 269,304 16S rRNA sequences generated a total of 6,867 OTUs. Rarefaction analyses (Figure S2) and Good’s coverage estimator suggested that the sequencing depth covered between 93.7 and 97.8% of the OTU richness within the samples. The highest bacterial diversity occurred in the Calcaric plots (H′ = 6.12 ± 0.02 for bulk soil, 6.16 ± 0.07 for rhizosphere soil; *P* < 0.05), with no significant differences between the compartments (Table S2). For the two other soil types, the estimates of the bacterial diversity decreased from Eutric to Hyperdystric plots, with higher H′ values in the rhizosphere compared to the surrounding bulk soil (*P* < 0.05; ANOVA) (Table S2). The bacterial richness (number of OTUs) followed the same trend, with a significant decrease from Calcaric to Hyperdystric plots (*P* < 0.05; ANOVA). At the phylum level, the multivariate analyses revealed shifts in the bacterial community structure between Calcaric plots and those of the two other soil types for both the bulk soil and rhizosphere compartments (Fig. 1A and B). This trend was confirmed by an Permutational Multivariate Analysis of Variance (PERMANOVA) analysis at the phylum level (P < 0.05) as well as at the OTU level (*P* < 0.05). When the compartments were considered independently, the bulk soil samples from the Eutric and Hyperdystric soils appeared significantly different at both class and OTU levels (Fig. 1A and Figure S3A; PERMANOVA *P* < 0.05), while the rhizosphere were not significantly differentiated (Fig. 1B and Figure S3B; PERMANOVA *P* > 0.05). When the two compartments (R and BS) were considered for each soil type, a rhizosphere effect was detected based on the multivariate (Fig. 2A,B and C) and PERMANOVA analyses (*P* < 0.05) at both class and OTU levels (Figure S3C,D,E).

#### GeoChip microarray

The GeoChip analysis revealed that a total of 49,867 bacterial probes (ranging from 38,531 to 44,173 on average) were detected, which corresponds to 709 different functional genes. Significantly higher gene diversity (H′) was observed for the bacterial communities occurring in the Eutric soils compared to those of the Calcaric soils (*P* < 0.05, ANOVA), while no significant difference in gene number was observed between the soil and rhizosphere compartments (Table S2). The same trend was observed for gene richness. A detailed analysis revealed that more than 92% of the genes, in terms of presence/absence, were shared between all samples across the toposequence (Figure S4). The multivariate analysis revealed that the bulk soil samples from the Hyperdystric and Calcaric plots were differentiated from those of the Eutric plots. However, only the difference between the Eutric and Calcaric bulk soil samples appeared significant (Fig. 1C; PERMANOVA, *P* < 0.001). A second multivariate analysis done only on the rhizosphere samples showed an overlap of all the samples regardless of the soil type considered (Fig. 1D). However, a PERMANOVA analysis revealed that the Eutric and Calcaric rhizosphere samples were significantly different (*P* < 0.001; Fig. 1D). When considered independently for each soil type, the rhizosphere and bulk soil compartments were separated (Fig. 2D,E and F), but the difference was non-significant based on a PERMANOVA analysis (P > 0.05).

### Comparison of bacterial community structure along the toposequence

**Figure 3:**
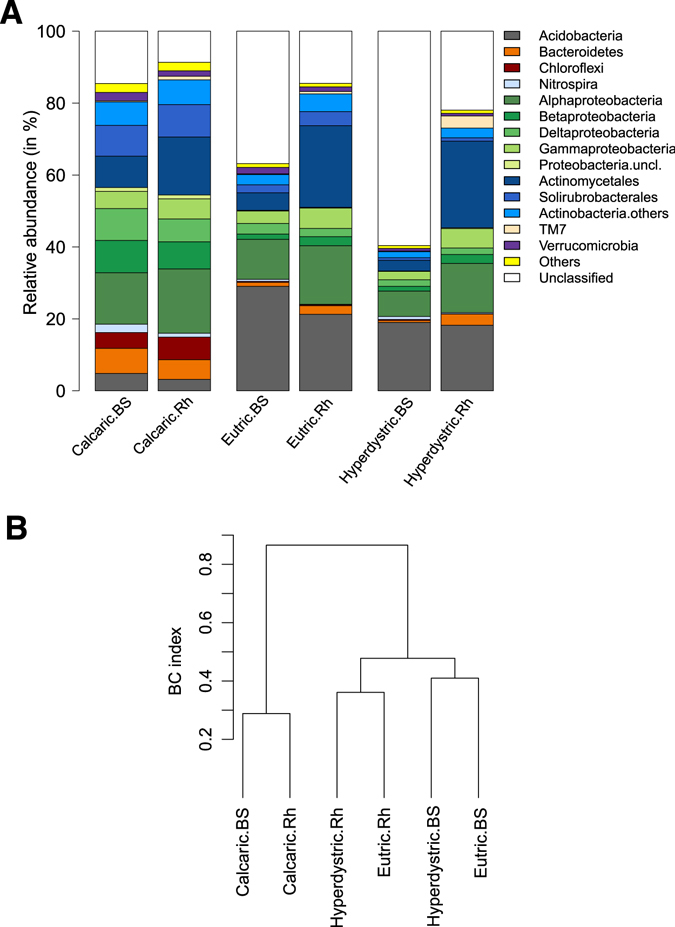
Distribution of the major bacterial phyla and classes along the toposequence and hierarchical clustering analysis. (**A**) Taxonomic distribution. The relative abundance of the major phyla and classes was calculated as the percentage of sequences belonging to a particular lineage of all 16S rRNA gene sequences recovered from each sample. The *Proteobacteria* phylum is represented in green and was detailed for the different classes. The *Actinobacteria* phylum was represented in blue and was detailed for the *Actinomycetales* and *Solirubrobacterales* orders. (**B**) Hierarchical clustering analysis. A hierarchical clustering based on Bray-Curtis (BC) distance of the major bacterial phyla and classes was performed. Samples are referred as follow: Calcaric.BS: Calcaric bulk soil; Calcaric.Rh: Calcaric rhizosphere; Eutric.BS: Eutric bulk soil; Eutric.Rh: Eutric rhizosphere; Hyperdystric.BS: Hyperdystric bulk soil; Hyperdystric.Rh: Hyperdystric rhizosphere.

The analysis of the 16S rRNA pyrosequences revealed that the toposequence was dominated by representatives of the phyla Proteobacteria (26.5%), Actinobacteria (21.7%) and Acidobacteria (15.9%), followed by those of Bacteroidetes (3.3%), Chloroflexi (1.9%), Verrucomicrobia (1.4%), Candidate Division TM7 groups (1%), Nitrospirae (0.9%), Gemmatimonadetes (0.7%) and Firmicutes (0.4%). Within the Proteobacteria, the main classes were the Alphaproteobacteria (13.4%), Gammaproteobacteria (4.5%), Betaproteobacteria (4.0%), and Deltaproteobacteria (4.0%) classes, while the Actinobacteria were mainly represented by the Actinomycetales (13.2%) and Solirubrobacterales (4.2%) orders. A detailed analysis of the relative distribution of the 16S rRNA gene sequences from the phylum to genus levels revealed significant differences across the toposequence based on the soil types and soil compartments (Fig. 3A, Tables 1, 2, S3 and S4).

**Table 1:** Significant variations in bacterial genera along the toposequence of Montiers.

Phylum	Genus	Bulk Soil				Rhizosphere			
		Calcaric	Eutric	Hyperdystric	p	Calcaric	Eutric	Hyperdystric	p
Actinobacteria	*Lamia*	1.80^a^ ± 0.10	0.73^b^ ± 0.22	0.45^b^ ± 0.13	***	1.83^A^ ± 0.11	1.08^B^ ± 0.11	0.84^B^ ± 0.14	***
	*Acidothermus*	1.39^a^ ± 0.15	0.01^b^ ± 0.01	0.00^b^ ± 0.00	***	1.44^A^ ± 0.41	0.03^B^ ± 0.02	0.00^B^ ± 0.00	**
	*Actinospica*	0.00 ± 0.00	0.13 ± 0.08	0.12 ± 0.07	0.305	0.02^B^ ± 0.01	3.18^B^ ± 0.32	7.84^A^ ± 2.26	**
	*Mycobacterium*	1.94^a^ ± 0.31	1.42^ab^ ± 0.50	0.41^b^ ± 0.24	*	3.58^AB^ ± 1.11	5.10^A^ ± 0.87	1.70^B^ ± 0.17	*
	*Nocardioides*	0.69^a^ ± 0.16	0.05^b^ ± 0.02	0.01^b^ ± 0.01	***	1.48^A^ ± 0.35	0.11^B^ ± 0.04	0.02^B^ ± 0.01	**
	*Strepacidiphilus*	0.00^a^ ± 0.00	0.06^a^ ± 0.02	0.06^a^ ± 0.03	0.09	0.01 ± 0.01	2.37 ± 0.33	5.84 ± 2.96	0.10
	*Streptomyces*	0.20^a^ ± 0.07	0.06^ab^ ± 0.03	0.02^b^ ± 0.01	*	1.32^A^ ± 0.28	0.47^B^ ± 0.19	0.22^B^ ± 0.13	*
	*Conexibacter*	4.65^a^ ± 0.50	1.56^b^ ± 0.52	0.45^b^ ± 0.19	***	4.86^A^ ± 1.18	2.28^AB^ ± 0.52	0.57^B^ ± 0.10	**
Gemmatimonadetes	*Gemmatimonas*	1.21^a^ ± 0.09	0.76^ab^ ± 0.18	0.42^b^ ± 0.13	**	0.89^A^ ± 0.16	0.39^B^ ± 0.05	0.25^B^ ± 0.07	**
Nitrospira	*Nitrospira*	2.30^a^ ± 0.18	0.54^b^ ± 0.19	0.84^b^ ± 0.11	***	1.08^A^ ± 0.15	0.13^B^ ± 0.03	0.33^B^ ± 0.05	***
Proteobacteria	*Bradyrhizobium*	0.98^ab^ ± 0.05	1.57^a^ ± 0.22	0.72^b^ ± 0.14	*	1.59^B^ ± 0.29	3.35^A^ ± 0.45	2.16^AB^ ± 0.48	*
	*Pedomicrobium*	1.02^a^ ± 0.07	0.02^b^ ± 0.01	0.03^b^ ± 0.01	***	0.74^A^ ± 0.10	0.04^B^ ± 0.02	0.02^B^ ± 0.01	***
	*Rhodoplanes*	2.15^a^ ± 0.11	0.37^b^ ± 0.08	0.09^b^ ± 0.03	***	2.21^A^ ± 0.27	0.62^B^ ± 0.07	0.18^B^ ± 0.06	***
	*Burkholderia*	0.01^b^ ± 0.00	0.12^a^ ± 0.03	0.15^a^ ± 0.03	*	0.03^B^ ± 0.01	0.81^A^ ± 0.16	1.05^A^ ± 0.25	**

All the data presented correspond to the mean relative abundance (±Standard Error of the Mean) of four independent replicates collected along the toposequence of Montiers. Only the genera accounting for more than 1% of total reads and varying significantly have been presented. For each genus, significant differences are presented by different letters (a b c for the bulk soil comparison; A B C for the rhizosphere comparison; ANOVA, P < 0.05). The asterisks correspond to the range of p value (‘*’ means the p-value < 0.05, ‘**’ means the p-value < 0.01, ‘***’ means the p-value < 0.001).

**Table 2:** Bacterial recruitment in the beech rhizosphere along the toposequence of Montiers.

Phylum	Genus	Calcaric			Eutric			Hyperdystric		
		Bulk soil	Rhizosphere	p	Bulk soil	Rhizosphere	p	Bulk soil	Rhizosphere	p
Actinobacteria	*Actinospica*	0.00 ± 0.002	0.02 ± 0.009	0.08	0.13 ± 0.082	3.18 ± 0.324	***	0.12 ± 0.071	7.84 ± 2.259	*
	*Catenulispora*	0.00 ± 0.000	0.00 ± 0.003	0.134	0.06 ± 0.024	0.64 ± 0.239	0.05	0.05 ± 0.019	0.77 ± 0.172	**
	*Dactylosporangium*	0.47 ± 0.056	0.80 ± 0.116	*	0.02 ± 0.009	0.30 ± 0.073	NaN	0.00 ± 0.002	0.06 ± 0.021	*
	*Mycobacterium*	1.94 ± 0.313	3.58 ± 1.107	0.205	1.42 ± 0.501	5.10 ± 0.866	*	0.41 ± 0.239	1.70 ± 0.174	**
	*Kineosporia*	0.00 ± 0.002	0.00 ± 0.002	1	0.00 ± 0.000	0.01 ± 0.004	1	0.02 ± 0.008	0.28 ± 0.076	*
	*Streptacidiphilus*	0.00 ± 0.000	0.01 ± 0.009	0.168	0.06 ± 0.022	2.37 ± 0.333	***	0.06 ± 0.025	5.84 ± 2.963	0.099
	*Streptomyces*	0.20 ± 0.068	1.32 ± 0.275	**	0.06 ± 0.03	0.47 ± 0.190	0.081	0.02 ± 0.005	0.22 ± 0.130	0.169
Firmicutes	*Bacillus*	0.70 ± 0.119	0.86 ± 0.213	0.535	0.00 ± 0.000	0.03 ± 0.010	*	0.01 ± 0.009	0.06 ± 0.037	0.289
Proteobacteria	*Phenylobacterium*	0.19 ± 0.012	0.29 ± 0.063	0.146	0.43 ± 0.115	0.59 ± 0.073	0.26	0.14 ± 0.013	0.34 ± 0.064	*
	*Bradyrhizobium*	0.98 ± 0.050	1.59 ± 0.286	0.081	1.57 ± 0.218	3.35 ± 0.453	*	0.72 ± 0.144	2.16 ± 0.479	*
	*Burkholderia*	0.01 ± 0.004	0.03 ± 0.008	0.092	0.12 ± 0.029	0.81 ± 0.157	**	0.15 ± 0.034	1.05 ± 0.246	*
	*Methylibium*	0.00 ± 0.004	0.01 ± 0.006	0.585	0.02 ± 0.009	0.12 ± 0.045	0.069	0.16 ± 0.018	0.47 ± 0.067	**
	*Collimonas*	0.00 ± 0.004	0.27 ± 0.124	0.08	0.02 ± 0.004	0.12 ± 0.040	*	0.00 ± 0.000	0.00 ± 0.000	NaN

All the data presented correspond to the mean relative abundance (±Standard Error of the Mean) of four independent replicates collected along the toposequence of Montiers. Only the genera accounting for more than 0.25% of total reads and that were significantly increased in beech rhizosphere have been presented. For each genus, the significant differences are indicated with asterisks (‘*’ means the p-value < 0.05, ‘**’ means the p-value < 0.01, ‘***’ means the p-value < 0.001).

#### Soil type effect

The hierarchical clustering analysis revealed a strong differentiation of the bacterial communities of the Calcaric samples from those of the Eutric and Hyperdystric soils (BC > 0.83; Fig. 3B). The structure of the bacterial communities appeared to be significantly impacted by the soil conditions occurring across the toposequence (Fig. 3, Tables 1 and S1). When considering the effect of soil type on only the bulk soil bacterial communities, it appears that those of the Calcaric plots were significantly enriched in the sequences related to the Proteobacteria, Firmicutes, Bacteroidetes, Chloroflexi and Nitrospirae phyla compared to the two other soil types (*P* < 0.05; Fig. 3A and Table S3). Significant differences were also observed at the class to order levels (Table S3). At the genus level, the most enriched representatives were assigned to the *Lamia*, *Acidothermus*, *Mycobacterium*, *Conexibacter*, *Pedomicrobium*, *Nitrospira* and *Rhodoplanes* genera (*P* < 0.05; Table 1). In contrast, in the Hyperdystric and Eutric plots, non-assigned sequences or those assigned to the Acidobacteria phylum were significantly enriched compared to the Calcaric plots (*P* < 0.05). A higher relative abundance of Acidobacteria sequences was observed in the Eutric plots (Fig. 3A). These Acidobacteria representatives were mainly affiliated with groups 1, 2 and 3. At the genus level, the most enriched representatives were assigned to the *Burkholderia* genus (Table 1). When considering the effect of soil type on only the rhizosphere bacterial communities, the same trends were observed for most of the phyla, classes, orders and genera (Table S3).

#### Rhizosphere effect

The hierarchical clustering analysis (Fig. 3B) also highlighted the differentiation of the bacterial communities based on their compartment of origin (i.e., rhizosphere or bulk soil), with a more pronounced rhizosphere effect in the Hyperdystric (R vs BS; BC = 0.48) and Eutric (R vs BS; BC = 0.40) soils than in the Calcaric (R vs BS; BC = 0.29) soil. In the Calcaric soil, most of the changes were related to a significant decrease of the relative abundance of the Acidobacteria, Bacteroidetes, Nitrospirae and Deltaproteobacteria in the rhizosphere, while the Caulobacterales, Rhodobacterales, Burkholderiales, and Rhodocyclales orders and the Kineosporiaceae, Micromonosporaceae and Streptomycetaceae families were significantly enriched (*P* < 0.05, ANOVA) (Table S4). Notably, among the assigned genera, *Streptomyces* showed the most significant increase in the rhizosphere (BS = 0.2% vs Rh = 1.3%) (Table 2). When considering the two acidic soil types, similar patterns were observed. In these soil types, a significant decrease in the relative abundance of Acidobacteria group 5, Chloroflexi and Nitrospirae was observed in the rhizosphere, while most of the Actinobacteria, Bacteroidetes, Firmicutes, Betaproteobacteria and Gammaproteobacteria were enriched (P < 0.05; Table S4). At the genus level, the sequences assigned to *Actinospica*, *Mycobacterium*, *Streptacidiphilus*, *Bradyrhizobium* and *Burkholderia* were significantly enriched in the rhizosphere (Table 2). The cumulated relative abundance of these 5 genera between the bulk soil and rhizosphere compartments ranged from 3.8 to 14% and from 1.3 to 17% in the Eutric and Hyperdystric plots, respectively (Table 2).

#### Rhizosphere core microbiome

**Figure 4:**
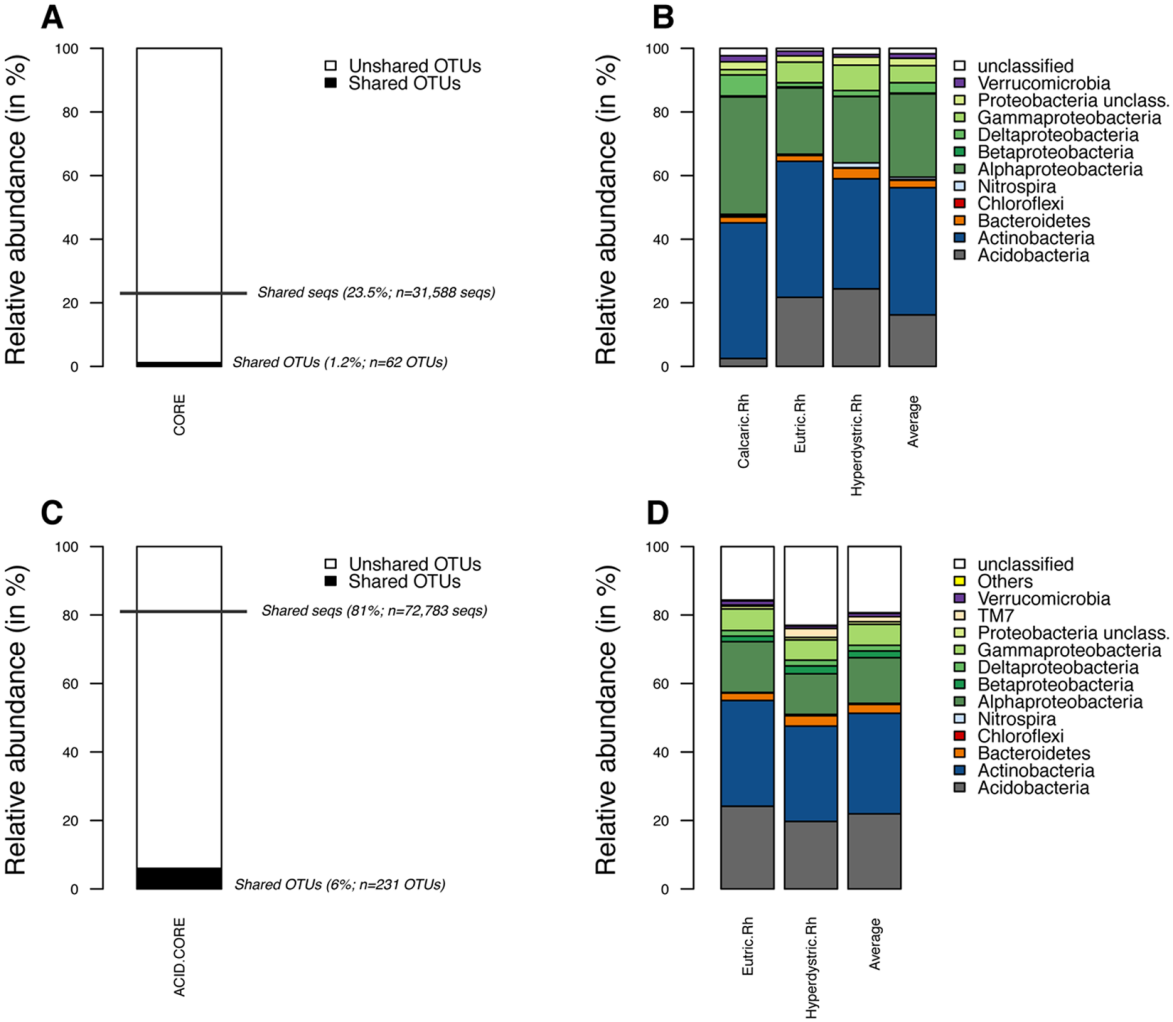
Characteristics of the rhizosphere/core microbiome. (**A**,**B**) Rhizosphere/core OTUs common to all the soil types of the toposequence. (**A**) The relative abundance of the core rhizosphere was calculated as the percentage of shared OTUs and shared sequences (shared seqs) among the three soil types (Calcaric, Eutric and Hyperdystric). (**B**) Taxonomic affiliation of the core rhizosphere and relative distribution in each soil type and in average view of the three soil types. (**C**,**D**) Rhizosphere/core OTUs common to the acidic soil types. (**C**) The relative abundance of the core rhizosphere was calculated as the percentage of shared OTUs and shared sequences among the two acidic soil types (Eutric and Hyperdystric). (**D**) Taxonomic affiliation of the core rhizosphere and relative distribution in each soil type and in average view of the two acidic soil types. For each analysis concerning the shared OTUs (**A** and **C**), the relative percentage and the number of OTUs are presented between brackets. For each analysis concerning the shared sequences (**A** and **C**), the relative percentage and the number of sequences are presented between brackets. Samples are referred as follow: Calcaric.Rh: Calcaric (Rhizosphere); Eutric.Rh: Eutric (Rhizosphere); Hyperdystric.Rh: Hyperdystric (Rhizosphere). For both shared OTUs (OTUs shared by the samples) and shared sequences (number of sequences corresponding to the shared OTUs), data are presented in relative abundance and as absolute value.

To determine if beech selects a core microbiome within the vicinity of its roots regardless of the soil type, a comparative analysis was performed at the OTU level. When the analysis was performed considering the OTUs present in all the rhizosphere samples and potentially present in one or more of the bulk soil samples, regardless of the soil type, a total of 62 OTUs (1.2% of all OTUs detected in the rhizosphere samples) were obtained, which represented 23.5% of the total reads generated for the rhizosphere samples (Fig. 4A). A detailed analysis revealed that the rhizosphere core microbiome was dominated in terms of relative abundance and OTU richness by the sequences assigned to the phyla Acidobacteria, Actinobacteria and Alphaproteobacteria and to the genera *Mycobacterium*, *Bradyrhizobium* and *Rhodoplanes* (Fig. 4B and Table S5).

Due to the important edaphic differences between the three soil types considered, the same analysis was performed between the two acidic soil types (Hyperdystric and Eutric). This analysis revealed that the acidic rhizosphere/core microbiome was characterized by a total of 231 OTUs (6% of all OTUs detected), which represented 81% of the total reads generated for the rhizosphere samples (Fig. 4C). This rhizosphere/core microbiome was characterized by similar phyla to those of the global core microbiome presented above, but the OTUs were assigned to additional genera such as *Steroidobacter*, *Streptacidiphilus*, *Actinospica*, *Mycobacterium* and *Bradyrhizobium* (Fig. 4D, Table S6).

### Analysis of detected functional genes across the toposequence

**Figure 5:**
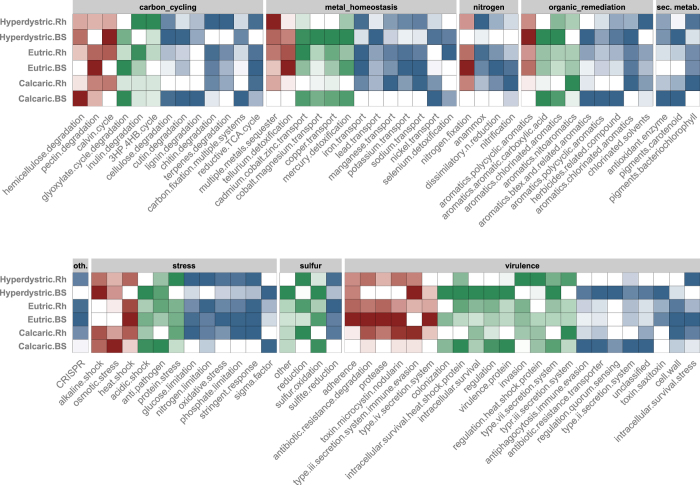
Heatmap presenting the functional subcategories varying significantly according to the soil type (red), between the bulk soil and the rhizosphere compartments (green), or overlapping soil type and compartment effects (blue). This Figure only presents the functional subcategories of the GeoChip microarray presenting a significant difference (P < 0.05) based on a One-factor ANOVA. Values were normalized so that the highest signal intensity of each subcategory is equal to 1 (dark colour) and the lowest is equal to 0 (white colour). For each function considered the colours from dark to white are separated according to 5 levels of intensity.

A detailed analysis of the GeoChip data revealed similar patterns in the different soil types regardless of the functional level considered (probe, gene, subcategories, categories) (Figure S5). For each sample, secondary metabolism and virulence constituted the two major functional categories and higher signal intensities were measured in the bulk soil compared to rhizosphere samples from the Calcaric and Hyperdystric soils (P < 0.05; Figure S5). In contrast, significantly higher signal intensities were observed for the functional categories related to metal homeostasis, the CRISPR (clustered regularly interspaced short palindromic repeats) system, stress and sulphur cycling in the rhizosphere compared to the bulk soil (P < 0.05, Figure S5). The analysis of the taxonomic information associated to these functional categories revealed that the higher signal intensities were assigned to the same five phyla/classes (Actinobacteria, Firmicutes, Alpha/Beta/Gamma-Proteobacteria), but in different order depending on the category (Table S7). For carbon cycling, metal homeostasis, secondary metabolism, stress, virulence and organic remediation categories, significantly higher signals were obtained for Actinobacteria in the bulk soil compared to rhizosphere samples (BS > R; P < 0.05, Table S7). Similar patterns were observed for genes derived from Alphaproteobacteria for the functional categories related to secondary metabolism and sulphur cycle (P < 0.05, Table S7). In contrast, higher signals were observed for probes assigned to Gammaproteobacteria in the rhizosphere than in the bulk soil for functions related to carbon cycling, metal homeostasis, stress, sulphur cycling and CRISPR system. Those analyses were completed at the subcategory level to decipher how the functional structure of the bacterial communities was affected by the soil type, soil compartment (i.e., rhizosphere vs. bulk soil) or the combination of both factors (Fig. 5). This analysis showed that at the subcategory level, most of the significant differences were related to a compartment effect or a combined soil type/compartment effect and rarely to a soil type effect alone. Although several functions were affected, we were mainly interested in the functions related to nutrient cycling, microbial interactions and secondary metabolism.

#### Nutrient cycling: Metal homeostasis and sulphur cycling

Most of the functions related to nutrient cycling were significantly structured based on the compartment of origin, except in the Eutric soil, which gave similar signal intensities for both the rhizosphere and bulk soil compartments (Fig. 5). Among the functions affected, those related to iron uptake showed a significantly higher intensity in the rhizosphere (Fig. 5). A detailed analysis revealed that this enrichment corresponded to higher signals for *cirA* (TonB-dependent iron-siderophore complex uptake receptor) assigned to *Sphingomonas*, *Ferrimonas and Alteromonas*, *feoB* (cytoplasmic membrane protein involved in ferrous iron uptake) assigned to *Paenibacillus* and *pvcC* (biosynthesis of pyoverdine) assigned to *Thermomicrobium* in the rhizosphere (Figures S6 and S7). Similarly, genes involved in the transport of potassium (*trkA*, *trkGH*), sodium (*nhaA*, *nhaB* and *nhaD*) and manganese (*psaA_5f0_Mn*, *mntH_Nramp*) were significantly enriched in the rhizosphere (Figure S6) and derived from bacteria related to the Actinobacteria (*Clavibacter*) and Alpha/Gamma/Delta-proteobacteria (*Desulfovibrio*, *Desulfotomaculum*, *Alishewanella*, *Magnetococcus* and *Xenorhabdus*) (Figure S7). In contrast, genes related to metal resistance and the transport of copper (*copA*, *cusC*), cobalt/nickel (*cnrC*), cadmium/cobalt/zinc (*czcD*) and nickel (*nicoT*, *nreB*) had a significantly higher intensity in the surrounding bulk soil (Fig. 5). For these genes, higher signal intensities were observed for probes assigned to Alpha/Gamma/Delta-proteobacteria (*Labrenzia*, *Methylobacterium*, *Escherichia*, *Enterobacter*, *Sagitulla* and *Pelagibaca*) (Figure S7). Considering sulphur cycling, a total of 17 functional genes were detected. Among them, genes involved in the oxidation and reduction of sulphur compounds varied significantly between the bulk soil and rhizosphere (*P* < 0.05, Fig. 5). Notably, genes related to sulphur reduction such as *cysJ* (NADPH-sulfite reductase alpha subunit) and *dsrA* (dissimilatory sulfite reductase alpha subunit) showed a significantly higher intensity in the rhizosphere than in the bulk soil, regardless of the soil type considered (*P* < 0.05). The probes associated to these genes were assigned mainly to Betaproteobacteria (*Neisseria*, *Burkholderia* for *cysJ*) and unidentified bacteria (for *dsrA*) (Figure S7). The opposite trend was observed for genes related to sulphur oxidation (*soxB*, *soxY*), which were derived from aerobic microorganisms (P < 0.05; Figure S7).

#### Microbial interactions

A total of 225 genes associated with bacterial virulence were detected in the toposequence, and some of them presented significantly different patterns (Fig. 5). A strict soil type effect was mainly observed for functions related to adherence, antibiotic, proteases and the production of toxins, which were enriched in the Eutric soils compared to the two other soil types. Concerning the rhizosphere effect, most of the genes related to colonization, intracellular heat shock proteins, regulation, virulence proteins, type II and VII secretion systems, anti-phagocytosis immune evasion, quorum sensing and antibiotic resistance transporters gave a higher signal in the bulk soil than in the rhizosphere in the Hyperdystric and Calcaric soils (*P* < 0.05; Fig. 5). A detailed analysis revealed that the decrease in the rhizosphere was related to several genes encoding efflux pumps (*mfs*, *mex*), secretion systems (*xcpz*, *esxA*), anti-phagocytosis (alg) or genes involved in the regulation of virulence factors (*hfq*, *hrpG*, *igaA*, *saeR*, *vip*). In contrast, genes related to invasion or to the regulation of heat shock proteins (*hspR*) appeared to be enriched in the rhizosphere compartment. These variations of signal intensities were associated to various bacterial taxa among the Alpha/Gamma-proteobacteria and Firmicutes (Figure S9). When considering the CRISPR system, which is defined as a defence mechanism against phage infection, a total of 20 out of 47 genes were found to vary significantly in our samples. Among them, 13 exhibited significantly higher signal intensities in the rhizosphere (P < 0.05). The *cas1* and *cas2* genes, encoding universal proteins involved in new phage-derived spacer acquisition, were significantly enriched in the rhizosphere of the Hyperdystric and Eutric soils, respectively (*P* < 0.05; Figure S8). The higher signal intensities for these genes were assigned to *Myxococcus* (*cas1*) and *Albidiferax* (*cas2*) (Figure S9). In addition, other genes required for the processing of primary CRISPR transcripts, including the genes encoding the signature proteins of Type I (*cas3*) and Type II (*cas9_csn1*) CRISPR, were also significantly enriched in the rhizosphere systems of the Hyperdystric and Calcaric soils (P < 0.05; Figure S8). The probes associated to these genes were assigned mainly to *Desulfovibrio* (*cas3*) and to *Azospirillum* and *Ruminococcus* (cas9_csn1) (Figure S9).

#### Carbon degradation and secondary metabolism

Among the subcategories related to carbon degradation, a total of 13 varied significantly among the soil types or compartments considered. When considering the effect of soil type, hemicellulose degradation gave a significantly higher signal in the Calcaric plot than in the other soil types. The other subcategories were mainly influenced by a compartment effect or a combined compartment/soil type effect. Notably, higher signals were observed for the degradation of cellulose, cutin, glyoxylate or lignin in the bulk soil compared to the rhizosphere in the Hyperdystric and Calcaric soils. These results were explained at the gene level, by higher signal intensities for the genes encoding exoglucanase, endoglucanase and cutinase (Figure S10). In contrast, the signal intensities of the subcategories linked to chitin (chitinase) and terpene degradation were significantly higher in the rhizosphere (Fig. 5). For the functions related to secondary metabolism, all the observed changes were related to compartment or compartment/soil type effects (*P* < 0.05; Fig. 5). Notably, for all these functional categories, the higher signal intensities were obtained for probes assigned to Actinobacteria and more especially to the *Streptomyces*, *Gordonia*, *Actinosynnema* genera (Figure S11).

### Variance partitioning and correlations

A variance partitioning analysis was performed on the Biolog, the 16S rRNA pyrosequence and GeoChip data considering the soil type and compartment (rhizosphere vs bulk soil) effects. Our analysis revealed that the percentage of variance explained by both the soil type and the compartment effects was higher for the 16S rRNA pyrosequence (cumulative variance 60.5%) and the GeoChip data (52.1%) than the Biolog data (23.3%) (Figure S12). A detailed analysis of the variance partitioning showed that the soil type effect (STE) was higher than the compartment effect (CE) for the 16S rRNA pyrosequence (STE = 43.3% vs CE = 17.2%) and the Biolog (STE = 15.3% vs CE = 8.0%) data. On the contrary, the compartment effect was higher than the soil type effect for the Geochip data (CE = 30% vs STE = 22.1%).

In parallel, comparisons of soil parameters, phylogenetic diversity, metabolic potentials and the GeoChip data were done for the bulk soil samples. These comparisons revealed significant correlations according to a Mantel test (Table 3) and independent Pearson correlation tests (Tables S8–S10). A detailed analysis revealed that most of the different bacterial phyla detected were highly influenced by edaphic factors (Table S8). The most abundant phyla, i.e., Proteobacteria, Actinobacteria and Acidobacteria, were highly correlated with the soil pH and nutrient availability (CEC). Within the Proteobacteria phylum, the Betaproteobacteria and Deltaproteobacteria classes were strongly correlated with most of the environmental parameters tested, in contrast to the Alphaproteobacteria. Concerning the metabolic potentials, significant correlations were obtained with the soil parameters (Table S9) and the phylogenetic diversity (Table S10). The highest correlations between the metabolic potentials and soil parameters or phylogenetic diversity were observed for the same substrates (alpha-cyclodextrin, glycogen, 2-hydroxybenzoic acid and putrescine), except for D-galacturonic acid, which was only correlated with the soil parameters. Concerning the GeoChip data, significant correlations were only obtained with the phylogenetic diversity (Table 3). The Nitrospirae, Acidobacteria and TM7 division members were the phyla that were the most correlated with the functional subcategories.

**Table 3:** Mantel tests relating to Bray Curtis distance dissimilarity matrices generated from Biolog EcoPlates, Environmental parameters, 16S pyrosequencing and GeoChip data.

Mantel tests	BS	Rh	all
Environmental parameters/Phylogenetic diversity	0.887***	nc	nc
Environmental parameters/GeoChip	0.060	nc	nc
Environmental parameters/Biolog EcoPlate	0.522**	nc	nc
Phylogenetic diversity/GeoChip	0.116	0.037	0.194*
Phylogenetic diversity/Biolog EcoPlate	0.709***	0.512***	0.242**
GeoChip/Biolog EcoPlate	0.009	−0.067	0.004

The significant differences are indicated with asterisks. ‘*’ means the p-value < 0.05, ‘**’ means the p-value < 0.01, ‘***’ means the p-value < 0.001. Mantel tests were performed independently for the bulk soil (BS) and rhizosphere (Rh) samples or mixed together (all). Correlation analyses including the soil parameters were only done on the BS samples, because soil parameters were only determined for the bulk soil samples. ‘nc’ means non calculated.

## Discussion

The impacts of soil parameters and plants on the taxonomic and functional structure of soil bacterial communities has been established, but studies have rarely addressed the combined impacts of soils and plants using non-perennial plants and never using trees. In this context, the experimental site of Montiers, which is characterized by a single dominant tree species and identical climatic conditions and forestry histories across a soil toposequence, gave us the first opportunity to test these combined effects in a set of different soil types covering acidic and nutrient-poor conditions to neutral and nutrient-rich conditions. Using a combination of soil analyses, metabolic assays, and phylogenetic and functional metagenomics, we tested the hypothesis that beech trees select specific bacterial taxa and functions within their root vicinity based on soil conditions and their own nutritional requirements.

The initial analysis of the bacterial communities inhabiting the different soil types revealed a significant differentiation of these communities based on the soil conditions. Indeed, the bacterial diversity, richness index and structure varied significantly across the toposequence, with a higher diversity in the neutral (pH = 7) and nutrient-rich soils (Calcaric) and a lower diversity in the acidic (pH = 4.5) and nutrient-poor soils (Hyperdystric). The importance of the soil type effect was also evidenced by the analysis of the partitioning of variance for the 16S rRNA pyrosequence data. In addition to the variation in the abundance of several bacterial taxa, significant correlations between soil parameters and the relative abundances of several of these taxa were also observed. Such relationships between diversity and pH or other soil parameters and the distribution patterns of several taxa across the toposequence confirmed the results of previous studies, which showed that specific taxa are enriched based on the soil pH and/or nutrient availability^38, 43, 46–48^. At the phylum level, the relative abundance of sequences assigned to Acidobacteria was significantly increased in the Eutric and Hyperdystric soils compared to the Calcaric soils. Representatives of this phylum are known to dominate acidic and nutrient-poor soils, suggesting an adaptation to oligotrophic conditions^38, 47, 49, 50^. In contrast, the relative abundance of sequences assigned to Proteobacteria, Chloroflexi and Bacteroidetes significantly increased in the Calcaric compared to the Eutric and Hyperdystric soils. The enrichment of these taxa in soils characterized by higher pH and nutrient contents fits very well with the copiotrophic lifestyle proposed by Fierer *et al*.^51^. Altogether, our results show that the reservoir of soil bacterial communities varies significantly based on the soil type across the toposequence.

In this context, we asked if beech trees selected a rhizosphere core microbiome common to all of the soil types or if specific taxa were selected based on the soil type. The concept of a core microbiome was initially developed in medicine and then for animals and plants with the idea that the microbiome played an important role in health and nutrition of its host^52–61^. More recently, the use of the core microbiome^61^ was proposed to better understand the similarities and dissimilarities in complex microbial assemblages and their roles in ecosystem functioning. Notably, most of the studies addressing humans have reported a very small core microbiome at the taxonomic level and a more important one at the functional level^52^. Indeed, at the taxonomic level, 0 to 0.5% of the OTUs appeared to be shared among samples^52, 55, 58^. The same trend was observed for fungal communities when comparing different soils^60^. Such low overlap is likely due to sequencing coverage efforts that were insufficient and the way that the OTUs were generated, resulting in high heterogeneity among the samples. In this sense, Ainsworth *et al*.^54^ showed that the size of the coral core microbiome ranged from 0.09% of the OTUs when considering the OTUs shared by 90% of the samples to 0.5% of the OTUs for those shared by 75% of the samples. To our knowledge, presence of a core microbiome for trees was only investigated by Shakya *et al*.^59^ on *Populus deltoides* trees by comparing the endosphere, the rhizosphere and the surrounding bulk soil. Their analysis revealed a small core rhizosphere microbiome represented by 35 bacterial OTUs, and a core endosphere microbiome represented by only a single OTU. In our study, the beech rhizosphere core microbiome represented 1.2% (62 OTUs; corresponding to 23.5% of the rhizosphere sequences) of the OTUs present in all the rhizosphere samples when the three soil types were considered together. When the same analysis was conducted using only the two acidic soil types, this value reached 6% of the OTUs (81% of the rhizosphere sequences), most likely due to the similarities between the edaphic parameters of the Eutric and Hyperdystric soils. Together, these results confirmed that the soil remains the reservoir of biodiversity from which specific taxa are enriched in the beech rhizosphere^62, 63^ and that a similar fraction of the soil bacterial communities is selected in the rhizosphere across all soil types.

Although the rhizosphere core microbiome represents a small fraction of the total diversity observed, our analyses revealed a rhizosphere effect regardless of the soil type considered. Indeed, several taxa such as Actinobacteria, Alphaproteobacteria, Betaproteobacteria and Gammaproteobacteria were significantly and selectively enriched depending on the soil type in the beech rhizosphere compared to the surrounding bulk soil. Such rhizosphere effects may be attributed in part to rhizodeposits and soil parameters, which may vary across the toposequence. Although beech root exudates were not analysed in our study, alterations to tree physiology and root exudation as a result of the soil conditions cannot be excluded. Indeed, plants are known to adapt their physiology, root architecture and root exudation to environmental conditions^1, 64^. In this study, we showed that the total carbon pool was significantly higher in the Calcaric compared to the Eutric and Hyperdystric soils. Preliminary results obtained in the bulk soil revealed that the same main carbohydrates were present in the bulk soil samples across all soil types. A survey of the literature suggests that plant exudation may vary quantitatively depending on the soil conditions and the plant nutritional status but not qualitatively^10, 63^. As an example, Neumann *et al*.^64^ showed that the same amino acids and carbohydrates were detected in the root exudates of lettuce regardless of the soil type, but that their concentrations varied quantitatively depending on the soil type. Notably, Schreiter *et al*.^65^ showed using these same soils that the rhizosphere effect was more or less pronounced depending on the soil type, suggesting a strong relationship with the soil parameters. Indeed, the soil parameters and especially the nutrient availability may be other parameters explaining the rhizosphere effect^18, 66, 67^. Although we were unable to measure the soil parameters of the rhizosphere in this study, a global analysis of the community structure variance revealed that the compartment effect explained 17% of the variance observed for the 16S rRNA pyrosequence data. This result suggests that chemical differences occur between the bulk soil and rhizosphere compartments. In this sense, previous measurements of forest soils and especially of beech soils have shown that nutrient concentration gradients between the rhizosphere and the bulk soil do occur, with the accumulation of nutritive cations (Ca, K, Mg) and carbon occurring in the rhizosphere^18, 19^. Because bacterial communities are strongly affected by soil parameters such as pH, nutrient availability and aluminium concentration^38, 39, 41, 43^, we cannot exclude that part of the rhizosphere effect on the bacterial communities may be due to the accumulation of nutritive cations in the rhizosphere. In this sense, we showed that the enrichment of specific taxa was significantly more pronounced in the most acidic and nutrient-poor soils than in the calcareous and nutrient-rich soils. This trend was confirmed by the Bray-Curtis analysis, which showed that the rhizosphere and bulk soil samples of the Calcaric soil were strongly related, in contrast to the Eutric and Hyperdystric rhizosphere and bulk soil samples, which were more differentiated. These results obtained for the first time under field conditions for trees fit very well with those obtained for lettuce^65^ or under controlled conditions for *Arabidopsis thaliana* ^57, 68^.

A detailed analysis revealed that specific bacterial genera were selectively enriched in the beech rhizosphere compared to the surrounding bulk soil, making them potential plant growth-promoting rhizobacteria (PGPR) or profiteers. Among the identified genera, most belonged to the Actinomycetales order. Their presence in forest soils was not unexpected, as they are known to decompose complex compounds, such as starch, pectin, lignin and chitin, and to produce antibiotic metabolites^69^. Notably, *Actinospica*, *Catenulispora*, *Mycobacterium* and *Streptacidiphilus* were significantly enriched in the beech rhizosphere in the two most acidic and nutrient-poor soils, in contrast to *Streptomyces*, which was significantly enriched only in the calcareous and nutrient-rich soils. Interestingly, antifungal and siderophore production and phosphate solubilization activities were particularly conserved among acidophilic actinomycetes such as *Streptacidiphilus* and *Actinospica* ^70, 71^. Other genera related to the Proteobacteria such as *Bradyrhizobium*, *Burkholderia*, and *Methylibium* appeared to also be significantly enriched in the beech rhizosphere, but only in the acidic soils. Notably, all these genera have been previously noted for their ability to colonize root systems and to consume exudates^21, 72^. Representatives of these genera have also been previously described in acidic forest soils in terms of their ability to fix nitrogen for *Bradyrhizobium* or to weather minerals for *Burkholderia* ^33, 73^. The presence of these different genera in acidic and nutrient-poor conditions, their enrichment in the rhizosphere, and the fact that they are known for their ability to consume complex molecules and root exudates and to access nutrients suggest that beech trees promote specific bacteria within their root vicinity with the potential to benefit tree health and nutrition.

Altogether, our results showed an important taxonomic differentiation of the bacterial communities across the toposequence and a selective enrichment of specific taxa in the rhizosphere compared to the surrounding bulk soil, suggesting an adaptation to the soil conditions. Considering this, we can ask how this selection is translated in terms of function or, in other words, how beech trees address variable genomic pools in maintaining the functions necessary for their nutrition and health. To answer these questions, we have used the GeoChip array technology. Although this technology is powerful, it permits only to detect variations of genes or taxa represented on the array. In this sense, functions for which no genes have been characterized can not be analysed. This is for example the case of the genes related to the mineral weathering function. The GeoChip approach remains adapted for describing the functional structure of the microbial communities. Our results based on the GeoChip analyses revealed that the same functional genes were detected across the toposequence in both of the compartments considered. These results suggest that although the genomic pool varies across the toposequence, similar functions carried by different organisms are selected. Such conservation, also known as functional redundancy, has been previously described in association with soil gradients^46, 74, 75^. Although the same genes were detected qualitatively, significant quantitative differences were observed depending on the soil type, the soil compartment or both of these factors. Notably, the analysis of the partitioning of the variance highlighted that the compartment effect (30%) was higher than the soil type effect (22.1%) for the GeoChip data. A detailed analysis revealed that functions related to nutrient cycling such as metal homeostasis, sulphur cycling and interactions with bacteriophages were significantly enriched in the rhizosphere bacterial communities, while functions related to virulence and secondary metabolism were significantly enriched in the bulk soil bacterial communities. These variations of functional structure came with significant variations of the signal intensities of probes assigned to Actinobacteria and Alphaproteobacteria, suggesting an important role of these taxa in the related functional categories. The GeoChip notably highlighted greater signal in carbon degradation for Actinobacteria probes assigned to the *Actinosynnema*, *Gordonia*, *Mycobacterium* and *Streptomyces genera*. Notably, the higher signal for probes assigned to *Streptomyces* observed in acidic condition corresponds probably to genes conserved among *Streptomyces* and *S*t*reptacidiph* *ilus* ^70, 71^. Indeed, specific *Streptacidiphilus* probes are absent on the Geochip, while the 16S rRNA amplicon sequencing approach clearly showed a transition from the Calcaric soil (rich in *Streptomyces*) to the Hyperdystric soil (rich in *Streptacidiphilus*). These two closely related genera have been shown to be effective at degrading various carbon substrates^70, 71^. Concerning the *Burkholderia* and *Bradyrhizobium* genera, which were among the most significantly impacted taxa across the toposequence based on the 16S rRNA amplicon sequencing approach, higher signals were observed for functional probes related to antibiotic resistance system (MFS_antibiotic, mex) and metal homeostasis (*cysJ, nicoT*).

In addition to the aspects related to nutrient cycling, our GeoChip analyses revealed a significant enrichment of genes involved in the immune system of bacteria against viruses in the rhizosphere bacterial communities compared to those in the bulk soil. Interestingly, similar observations have also recently been found in the rhizosphere of barley, suggesting that this may be a common trait of rhizosphere bacterial communities^76^. The presence of defence systems against phage invasion has been described in bacteria and archaea^77^. In the arms race between prokaryotes and phages, the main known defence systems that have been developed by prokaryotes are related to the modification of receptors, abortive infection or the production of restriction endonucleases. In our study, several genes related to the newly described CRISPR-Cas (CRISPR-associated) system, such as *cas*, *cmr*, *csc*, *csx* and *csy*, appeared to be significantly enriched in the rhizosphere compartment. Regardless of the soil type considered, the highest signals were obtained for csy2, cas7 and cmr4. For most of these genes, the higher signals were obtained for probes assigned to Alpha-, Beta- and Deltaproteobacteria confirming the predisposition of Proteobacteria to develop resistance against phages observed by genome analysis^78^. Notably, all these genes usually encode RNA-binding proteins such as nucleases and helicases, allowing bacteria to incorporate DNA sequences derived from viruses into their genome^78^. The incorporated viral sequences are transmitted to progeny and prevent infection by the same virus. Based on the enrichment of CRISPR-Cas genes in the rhizosphere bacterial communities, we can ask if it results from positive selection applied to rhizosphere bacteria due to a high concentration of viruses in the rhizosphere or if it corresponds to the preferential development of previously infected bacterial taxa or from horizontal transfer. In our study, the probes of the GeoChip that targeted virus-related genes showed that most of the probes gave similar signals regardless of the soil type or compartment, although some appeared to be enriched in the rhizosphere or bulk soil compartments. Consistent with this, Swanson *et al*.^79^ reported that the virus-to-bacteria ratio was significantly reduced in the wheat rhizosphere compartment compared to that in the surrounding bulk soil, suggesting that the ability to resist phage attack may be an important ecological trait of the rhizosphere bacterial communities.

## Conclusion

In conclusion, the soil experimental site at Montiers proved to be a particularly relevant field site to test if members of the same plant species of the same age growing under similar climatic conditions but in different soil types promote specific bacterial taxa and functions in their rhizosphere depending on the soil conditions. Although such questions have been addressed under controlled conditions for *Arabidopsis thaliana* and barley^57, 68, 76^ or under field conditions for lettuce^65^, our study provides the first comprehensive view of the taxonomic and functional structure of the beech rhizosphere microbiome in relation to variable soil conditions. Our 16S rRNA gene amplicon-based pyrosequencing analyses revealed on one hand the existence of a conserved rhizosphere core microbiome across the toposequence and on the other hand a stronger rhizosphere effect at the taxonomic level in the acidic and nutrient-poor soils than in the neutral and nutrient-rich soils. This stronger rhizosphere effect may be related to a stronger investment of beech trees in the promotion of a microbiome capable of improving their health and nutrition, potentially through root exudation. In addition to the important taxonomic differentiation observed across the toposequence, our GeoChip analyses revealed relative functional redundancy. Although the GeoChip analysis did not provide an exhaustive view of the functional diversity, our new data give us a new picture of the complex interactions that exist between trees and microorganisms. Our results suggest that beech trees promote different taxa depending on the soil reservoir, but that carry similar functional genes. However, although our taxonomic and functional analyses revealed the selection of similar functions regardless of the soil type, they did not reveal how these functions are expressed and if the chemical composition of root exudates change. As such, metatranscriptomic and root exudate analyses will be necessary to delve deeper into the interactions among tree microorganisms and to determine if beech trees control the expression of bacterial functions based on their nutritional requirements.

## Materials and Methods

### Site description

The study was carried out at the long-term observatory (LTO) of Montiers (48.53N, 5.32E) in the Meuse department (in north-eastern France) and is part of the SOERE F-ORE-T (Environmental Research Monitoring and Experimentation Systems) and the European Union’s Infrastructure for Analysis and Experimentation on Ecosystems (AnaEE) networks. This experimental site is co-managed by the ANDRA (French national radioactive waste management agency), the Permanent Environmental Observatory (OPE) (ANDRA OPE) and the INRA (UMR1138 Unit; http://www.nancy.inra.fr/en/Outils-et-Ressources/montiers-ecosystem-research). The LTO is characterized by a soil sequence (SS2), previously described in Jeanbille *et al*.^43^, that covers a distance of ca. 1.5 km and is composed of three soil types. Due to the size of the soil sequence, each soil type is characterized by the same climatic conditions. The bedrock is composed of two geological layers: a Jurassic calcareous (Tithonian) layer overlaid by detrital sediments from the lower Cretaceous (Valanginian), which is mostly present at the top of the soil sequences. According the World Reference Base (IUSS Working Group, W. R. B., 2006), the soil sequence (SS2) is characterized by an upper plot characterized as a Hyperdystric Cambisol (altitude of ca. 400 m), a middle plot corresponding to a Eutric Cambisol and a lower plot corresponding to a Calcaric Cambisol (altitude of ca. 320 m; directly developed on a Tithonian limestone). For legibility reasons, the soil types have been designated Hyperdystric, Eutric and Calcaric throughout the manuscript. Due to forestry practices, the soil sequence is dominated by European beech trees (*Fagus sylvatica L*.), which represents 88% of the tree stand. All the beech trees of the Montiers experimental site are of similar age with an average age of 50 years in 2010. Consequently, this soil sequence represents the natural evolution of the same mineral parental material, giving us the opportunity to examine the impacts of the soil parameters on the soil and rhizosphere bacterial communities.

### Soil processing and analysis

Soil and rhizosphere samples were collected in October 2014. Sampling was performed in autumn because previous experiments have shown that this is the season during which the beech rhizosphere bacterial communities are more effective at mobilizing inorganic nutrients than those of the surrounding bulk soil^9, 18^. For beech trees, autumn is also the season when the carbohydrates produced by photosynthesis are progressively stored in roots and are expected to be transferred in large amounts to the rhizosphere compartment^80, 81^. Consequently, the beech rhizosphere compartment in autumn thus constitutes a microbial habitat where intense microbial activities take place. In each soil type, four spatially distant soil cores with 33 × 33 × 20-cm dimensions (after the removal of the litter layer) were collected. The soil samples were collected ten metres apart from one another under only mono-specific plots of beech along the toposequence to avoid the effects of mixed tree species. Only the 5–20-cm soil horizon was used in this study. This soil depth was selected for two reasons: (i) because it corresponds to the soil zone where most of the fine roots of trees, which are involved in tree nutrition, are concentrated^82–84^ and (ii) because the depth of the soil in the Montiers LTO varies from 20 cm in the Calcaric soil to a few metres in the Hyperdystric soil. For each soil core collected under a mono-specific plot, the woody roots with adhering soil were carefully separated from the surrounding bulk soil by gentle manual collection, resulting in a total of 12 bulk soil samples and 12 rhizosphere samples (24 samples in total). Beech roots can be unambiguously recognized *in situ* because of their colour, the presence of lignin and the absence of other plants in each plot. One hundred grams of non-adhering bulk soil and ten grams of roots and adhering soil from each soil core were collected and independently mixed for each compartment prior to enzymatic and molecular analysis. The remaining bulk soil samples were sieved (using a 2-mm mesh) and dried at 30 °C before soil analyses at the Laboratoire d’Analyse des Sols d’Arras (http://www5.lille.inra.fr/las). Due to the low amount of rhizosphere soil collected, soil analyses were only performed on the bulk soil samples. The cation exchange capacity (CEC) was determined using the cobaltihexamine method. Titration of the cobaltihexamine chloride soil extract was performed at 472 nm and compared to a reference 005 N cobaltihexamine chloride extract. The pH was determined by the water method using a soil:water ratio of 1:5 (w/v). Total carbon (C) and total nitrogen (N) contents (both obtained after combustion at 1000 °C) and phosphorus (P) content were determined^85–87^. Exchangeable cations (Ca, Mg,Na, K, Fe, Mn and Al) and H+ were extracted using cobaltihexamine and determined by inductively coupled plasma spectrometry-atomic emission spectrometry (ICP-AES) for cations and bypotentiometric measurement using 0.05 M KOH for protons.

#### Substrate utilization assays

Although the BIOLOG method is known for its biases (culture conditions, incubation time, …), ECOplates have been designed to determine the potential of microbial substrate utilization under similar conditions. In our case, EcoPlates (Biolog®) were used on the different soil type and compartments considered. For each sample, 5 grams of rhizosphere and bulk soils were suspended in 25 mL sterile distilled water, vortexed twice for 1 min and diluted at 1/20. The plates were incubated at 25 °C, and colour development was measured at 595 nm with an iMark microplate absorbance reader (Bio-Rad) after a 48-hour incubation period. The absorbance data were processed as recommended by the manufacturer and according to Lohmus *et al*.^88^.

### DNA extraction, amplification of 16S rRNA gene and pyrosequencing analyses

Genomic DNA was extracted from 5 g of homogenized bulk soil and root-adhering soil using the ‘PowerMax Soil^TM^ DNA Isolation Kit’ (MoBio Laboratories, Carlsbad, CA, USA) as recommended by the manufacturer. The 16S rRNA gene amplicon libraries were generated in one step, using the primers 799 F and 1115R^89, 90^ containing the specific Roche 454-pyrosequencing adaptors and 5 bases barcodes. Notably, these primers have been designed to avoid chloroplast DNA amplification. PCRs contained 1X PCR Mastermix (5 PRIME®), 500 nM 799 f primer, 500 nM 1115r primer and 8 ng of DNA in a final volume of 50 µL. Amplifications were performed using the following cycle parameters: 95 °C for 5 min (initial denaturation), followed by 30 cycles of 95 °C for 30 s, 57 °C for 35 s, and 72 °C for 30 s with a final extension step at 72 °C for 10 min. Triplicate PCR products were checked by gel electrophoresis for each sample, pooled and purified by using the QIAquick purification kit (Qiagen, Valencia, CA, USA) as recommended by the manufacturer. Concentration of each purified PCR product was measured using a Nanodrop-1000 spectrometer (NanoDrop Technologies, Wilmington, DE, USA) and an equimolar mix of the 16S rRNA gene amplicons was used for pyrosequencing on the Genome Sequencer (GS) FLX 454 Titanium platform (Roche) at the Beckman Genomic Coulter (Danvers, MA, USA). A total of 415,320 16S rRNA rawdata sequences were obtained after sequencing. Reads were filtered for length (>300 bp), quality score (mean, ≥25), number of ambiguous bases (=0), and length of homopolymer runs (<8) using the trim.seqs script in Mothur v.1.30.2^91^. After trimming, chimera and non bacterial sequence (mitochondria, chloroplasts, archaea, eukaryota) removal, a total of 332,335 16S rRNA sequences were obtained and were then aligned with the SILVA alignment and the operational taxonomic units (OTUs) were defined with 3% dissimilarity level. Chimeric sequences were detected using the chimera.uchime command and were removed from further analysis. Singletons were conserved in the analysis. To avoid any biases associated with different numbers of sequences in each of the samples, a randomly subsampled a total of 11,221 sequences (corresponding to the smaller set of sequences after MOTHUR processing) from each sample was performed giving a total of 269,304 16SrRNA gene sequences used for further analyses. Good’s coverage estimate was calculated using Mothur. Taxonomy was assigned to each OTU by aligning sequences against the SILVA alignment database with a bootstrap value of 80 for taxonomic assignment. To highlight the effects of the soil type and/or of the compartment (rhizosphere vs bulk soil), taxonomic analyses were presented all along the manuscript from the phylum to the genus level. Such way of presentation permits to highlight the variations of structure at different taxonomic levels and to consider unknown or candidate taxa that only affiliated at the family, order or phylum levels.

### GeoChip analysis

To determine the functional structure of the bacterial communities occurring across the toposequence GeoChip 5.0 (180 K) have been used. The GeoChip arrays contain about 167,044 distinct probes covering ca 395,000 coding sequences from more than 1,590 functional genes involved in microbial carbon, nitrogen, sulfur, and phosphorus cycling, energy metabolism, metal homeostasis, organic remediation, among other categories. However, as the probes on the microarrays are derived from a chosen set of genes/sequences that do not necessarily represent the known diversity of the microbial communities of the Montiers site, the GeoChip method can not give an exhaustive view of the diversity occurring in this experimental site as it fails to detect taxa not represented on the microarrays. Microarrays are well adapted for describing the functional structure of the microbial communities.

Three replicates (n = 3) from each plot and soil compartment (bulk soil and rhizosphere; n total = 18) selected at random and according to the DNA quality criteria (concentration and ratio 230/260 nm and 260/280 nm) were analyzed with GeoChip 5.0 (180 K). All procedures were performed at Glomics Inc. (Norman, Oklahoma, USA). Detailed protocols are presented in supplementary material. Briefly, DNA (20 ng) was amplified using the Templiphi kit (GE Healthcare) with a modified buffer^92^. Amplified DNA (~2 µg) was labelled using random primers and Klenow, cleaned using a QIAquick purification kit (Qiagen), and dried down in a SpeedVac (45 °C, 45 min; ThermoSavant). Labeled DNA was rehydrated with 27.5 µL deionized water, then 99.4 µL hybridization solution and then was hybridized for 20–22 hr at 67 °C plus 10% formamide as described previously^93^. After hybridization, slides were washed and then imaged with a NimbleGen MS 200 microarray scanner. The GeoChip data were then extracted using the Agilent Feature Extraction program and loaded onto the GeoChip data analysis pipeline (ieg.ou.edu/microarray/). Data normalization and quality filtering were performed with multiple steps^94, 95^. Spots were scored as positive and retained if the signal-to-noise ratio [SNR = (signal mean – background mean)/background standard deviation] was ≥2.0, the coefficient of variation (CV) of the background was <0.8, and the signal intensity was at least 250. Spots that were detected in only one of the biological replicates were removed. Logarithmic transformation was carried out for the remaining spots, and the signals of all spots were transferred into relative abundances. These data were used for the statistical analyses including calculation of the Shannon index. In our analysis only the bacterial probes have been considered.

### Statistical analysis

The impact of the ecological origin (soil type and compartment) on the Biolog data, the 16S rRNA pyrosequence data and the GeoChip data were determined by analysis of variance (one- and two-factor(s) ANOVA) with a Bonferroni-Dunn correction on each substrate/taxa/probe. The Bonferroni-Dunn correction is known to reduce the Type I Error rate when testing multiple hypotheses and to be one of the most stringent correction procedure comparatively to other corrections based on the computing of false discovery rates (FDRs). It was selected to avoid considering substrates/phyla/probes presenting weak differences among the 18 two to by two comparisons. For the Biolog assays, only the optical densities (OD 595 nm) higher than 0.1 have been used to perform ANOVA. The same values were also used to calculate the Shannon-Weaver index (H) as follows: H = −Σpi(ln pi), where pi is the ratio of the activity of each substrate (ODi) to the sum of the activities on all the substrates (ΣODi). For the 16S rDNA pyrosequence and GeoChip data sets, the relative abundances of taxa (from OTU to class) and of functional categories were transformed using the arcsine square root to achieve a normal distribution and to allow the analysis of variance (ANOVA). The taxonomic and functional richness based on the observed data (i.e. OTU and functional categories observed) and the Shannon-Weaver index were calculated using the vegan package^96^. A Bray-Curtis (BC) dissimilarity matrix was calculated for the 16S rRNA libraries and for the GeoChip data to allow Permutational Multivariate Analysis of Variance (PERMANOVA) and hierarchical cluster analysis with the unweighted pair-group average algorithm (UPGMA). For 16S rRNA pyrosequence data analyses were performed at different classification levels, from OTU to class. The impact of the soil type on the soil chemical properties was determined by analysis of variance (one-factor ANOVA) followed by Bonferroni-Dunn tests. The FactoMineR package^97^ was used to perform all the multivariate analyses on the pyrosequencing, Biolog and GeoChip data. For pyrosequencing and GeoChip data relative abundances have been used. A variance partitioning analysis was performed for the Biolog assays, the 16S rRNA pyrosequence data and the GeoChip data using the *varpart* command of the Vegan package, confirmed by an ANOVA analysis followed by ni-Dunn tests. Mantel tests (1,000 permutations, Pearson correlations) were performed to test correlations between soil parameters, Biolog data, pyrosequencing and GeoChip data based on Bray Curtis dissimilarity matrices generated from scaled data. Analysis of variance, multivariate analyses and Mantel tests were performed using R 3.1.1 software (R Core Team, 2014).

### Data Accessibility

The 454 pyrosequencing data generated for this study were submitted to the Sequence Read Archive (SRA) and are available under the Bioproject ID: PRJNA270036 and accession numbers SAMN04103543- SAMN034103566. The microarray data presented here are available for download at http://ieg.ou.edu/4download/.

## Electronic supplementary material

Supplementary material
