# Supplementary material for: Taxonomic and functional shifts in the beech rhizosphere microbiome across a natural soil toposequence
Source: Sci Rep. 2017 Aug 29;7:9604. doi: 10.1038/s41598-017-07639-1 (PMC5574896; doi:10.1038/s41598-017-07639-1)
Supplement: Supplementary file 1 — Supplementary material [file 41598_2017_7639_MOESM1_ESM.pdf]

SUPPLEMENTARY MATERIALS AND METHODS

Title: Taxonomic and functional shifts in the beech rhizosphere microbiome across a natural soil toposequence

AUTHORS: Colin, Y.[1,2], Nicolitch[1,2],O., Van Nostrand[3], J.D., Zhou[3,4,5], J.Z., Turpault, M-P.[1,2], Uroz, S.[1,2*]

[1] INRA, Université de Lorraine, UMR 1136 "Interactions Arbres Micro-organismes", Centre INRA de Nancy, 54280 Champenoux, France

[2] INRA UR 1138 "Biogéochimie des Ecosystèmes Forestiers", Centre INRA de Nancy, 54280 Champenoux, France.

[3] Institute for Environmental Genomics, and Department of Microbiology and Plant Biology, University of Oklahoma, Norman, OK 73072, USA.

[4] Earth Sciences Division, Lawrence Berkeley National Laboratory, Berkeley, CA 94720, USA.

[5] State Key Joint Laboratory of Environment Simulation and Pollution Control, School of Environment, Tsinghua University, Beijing 100084, China.

Running title: Beech rhizosphere microbiome

* Corresponding author: Mailing address: INRA-Université de Lorraine, Interactions Arbres Micro-organismes, UMR 1136, 54280 Champenoux, France. Phone: +33 (0)3 83 39 40 81, Fax: +33 (0)3 83 39 40 69. E-mail: uroz@nancy.inra.fr

GeoChip analysis

A total of three replicates (n=3) coming from each plot and each soil compartment (bulk soil and rhizosphere; n total=18) were used to perform GeoChip analyses. The gene array used (180K GeoChip 5.0) contains 167,044 distinct probes targeting functional genes involved in microbial carbon (degradation, fixation, methane), nitrogen, sulfur, and phosphorus cycling, energy metabolism, metal homeostasis, organic remediation, "Other" (phylogenetic genes and CRISPR system), secondary metabolism (e.g. antibiotic metabolism, pigments), stress responses, viruses (both bacteriophages and eukaryotic viruses), and virulence. All procedures were performed at Glomics Inc. (Norman, Oklahoma, USA). For each replicate considered, a total of 20 ng of DNA of the required quality (A260:A280 = 1.8; A260:A230 = 1.7) was amplified using the Templiphi kit (GE Healthcare) with the following modifications. Spermidine (0.1 mM) and single stranded binding protein (267 ng.mL^{-1}) were added to improve the amplification efficiency (Wu et al., 2006). Reactions were scaled down to 10 µL of sample and reaction buffers and 0.6 µl of enzyme. Samples were amplified for 6 hr. Amplified DNA (~2 µg) was mixed with 5.5 µL random primers (Life Technologies, random hexamers, 3 µg/µL), brought to 35 µL with nuclease-free water, heated to 99°C for 5 min, and immediately placed on ice. The labeling master mix {15 µl [2.5 µl of dNTP (5 mM dAGC-TP, 2.5 mM dTTP), 0.5 µl of Cy-3 dUTP (25 nM; GE Healthcare), 1 µl of Klenow (imer; San Diego, CA; 40 U.mL-1), 5 µL Klenow buffer, 2.5 µL of water]} was added and the samples were incubated at 37°C for 6 h in a thermocycler and then at 95°C for 3 min to inactivate the enzyme. After the addition of Cy3, samples were protected from the light as much as possible. Labeled DNA was cleaned using a QIAquick purification kit (Qiagen) per the manufacturer's instructions and then dried down in a SpeedVac (45°C, 45 min; ThermoSavant). Labeled DNA was rehydrated with 27.5 µL deionized water and then hybridization solution was added [99.4 µL; 63.5 µL 2X Hi-RPM hybridization buffer (Agilent), 12.7 µL pre-prepared 10X CGH blocking agent (Agilent), 12.7 µL formamide, 5.5 µL Cot-1 DNA, common oligo reference standard (Liang et al., 2010), mixed well, spun to collect liquid in bottom of tube, and incubated at 95°C for 5 min. Tubes were incubated at 37°C for 30 min. Gasket slides were placed into a SureHyb chamber, the samples (120 µL) were loaded onto the gasket slide. Samples were hybridized for 20-22 hr at 67°C. After hybridization, slides were disassembled in room temperature Wash Buffer 1 (Agilent) and then dried. GeoChips were imaged (NimbleGen MS 200 microarray scanner) as a Multi-TIFF. The GeoChip data were then extracted using the Agilent Feature Extraction program and loaded onto the GeoChip data analysis pipeline (ieg.ou.edu/microarray/). Data normalization and quality filtering were performed with multiple steps (Liang et al., 2010; Deng and He, 2014). First, the average signal intensity was calculated for each array, and the maximum average value was applied to normalize the signal intensity of samples in each array. Second, the sum of the signal intensity of samples was calculated for each array, and the

maximum sum value was applied to normalize the signal intensity of all spots in an array, which produced a normalized value for each spot in each array. Spots were scored as positive and retained if the signal-to-noise ratio [SNR = (signal mean – background mean)/background standard deviation] was ≥ 2.0, and the coefficient of variation (CV) of the background was < 0.8. In addition, spots with signal intensity less than 250 were discarded. Spots that were detected in only one of the biological replicates were also removed.

Deng Y, He Z. (2014). Microarray Data Analysis. In: He Z (ed) Microarrays: Current Technology, Innovations and Applications. Horizon Scientific Press: Norwich, UK).

Liang, Y.T., Z. H. He, L. Y. Wu, Y. Deng, G.H. Li, and J.-Z. Zhou. (2010). Development of a Common Oligonucleotide Reference Standard for microarray data normalization and comparison across different microbial communities. Appl. Environ. Microbiol, 76(4):1088-94. doi:10.1128/AEM.02749-09

Wu, L., Liu, X., Schadt, C.W., and Zhou, J. (2006) Microarray-based analysis of subnanogram quantities of microbial community DNAs by using whole-community genome amplification. Appl Environ Microbiol 72: 4931–4941.

Accession number

The 454 pyrosequencing data generated for this study were submitted to the Sequence Read Archive (SRA) and are available under the Bioproject ID: PRJNA270036 and accession numbers SAMN04103543- SAMN034103566. The microarray data presented here are available for download at http://ieg.ou.edu/4download/

Figure.S1 Principal component analysis (PCA) of the carbon substrates utilization pattern by total microbial communities. (A) PCA-ordination plots. PCA-ordination plots have been generated for the bulk soil and rhizosphere microbial communities based on their substrate utilization profile. The origins of samples are indicated as follows: filled squares, bulk soil samples from Cambisol Calcaric; open squares, rhizosphere samples from Cambisol Calcaric; filled circles, bulk soil samples from Cambisol Eutric, open circles, rhizosphere samples from Cambisol Eutric; filled triangles, bulk soil samples from Cambisol Hyperdystric; open triangles, rhizosphere samples from Cambisol Hyperdystric. Ellipses correspond to 95% confidence intervals about the mean. B) Vectors show the direction of maximum change for variables and longer arrows indicate a greater change in substrate utilization value. Substrates abbreviations used are: PAME, pyruvic acid methyl ester; tween 40, tween 40; tween 80, tween 80; a-cyclodextrin, alpha-cyclodextrin; glycogen, glycogen; D-cellubiose, D-cellubiose; a-D-Lactose, alpha-D-Lactose; BMetDGlc, beta-methyl-D-glucoside; D-xylose, D-xylose; i-Erythritol, i-Erythritol; D-Mannitol, D-Mannitol; NADG, N-Acetyl-D-Glucosamine; DGlcA, D-Glucosaminidic acid; Glc1P, Glucose 1 phosphate; GP, Glycerol phosphate; DGAGL, D-galactonique acid gamma-lactone; DGA, D-galacturonic acid; 2HBA, 2-Hydroxy-benzoic acid; 4HBA, 4-Hydroxy-benzoic acid; HbutA, Hydroxybutiric acid; Itaconic acid, Itaconic acid; ketobutyric acid, ketobutyric acid; malic acid, malic acid; arginine, arginine; asparag, asparagine; phenylala, phenylalanine; serine, serine; threonine, threonine; glutamic acid, glutamic acid; phenylethyl, phenylethylamine; putrescine, putrescine;

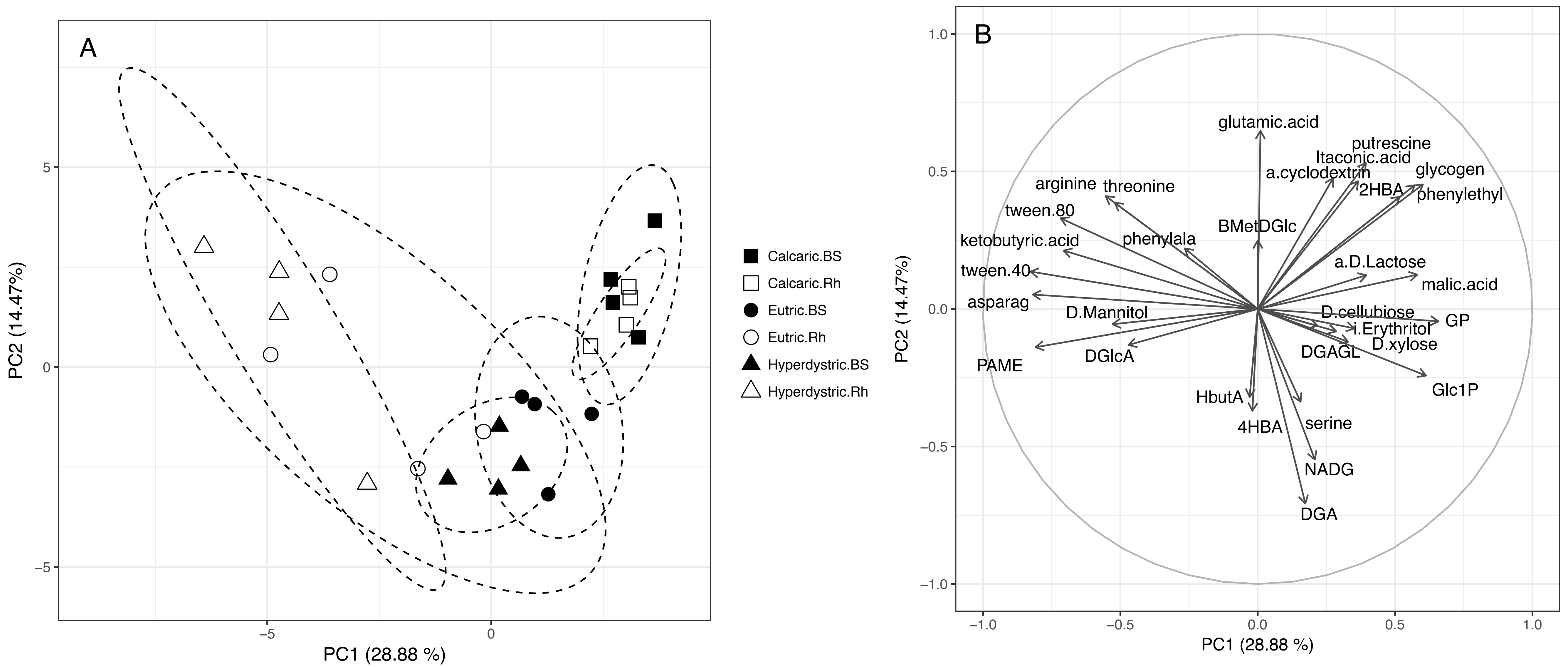
A
Calcaric.BS
Calcaric.Rh
Eutric.BS
Eutric.Rh
Hyperdystric.BS
Hyperdystric.Rh
PC1 (28.88 %)
PC2 (14.47%)
B
glutamic.acid
putrescine
Itaconic.acid
a.cyclodextrin
glycogen
2HBA
phenylethyl
arginine
threonine
tween.80
BMetDGlc
ketobutyric.acid
phenylala
a.D.Lactose
tween.40
malic.acid
asparag
D.Mannitol
D.cellubiose
GP
i.Erythritol
D.xylose
DGAGL
PAME
DGlcA
Glc1P
HbutA
4HBA
serine
NADG
DGA
PC1 (28.88 %)
PC2 (14.47%)

Figure.S2 Rarefaction curves indicating the number of operational taxonomic units (OTUs) at a genetic distance of 3% in Cambisol Calcaric (A), Eutric (B) and Hyperdystric (C) soils. The full and dotted lines refer to the rhizosphere and bulk soil samples, respectively. Samples are referred as follow: Calcaric.BS: Cambisol Calcaric bulk soil; Calcaric.Rh: Cambisol Calcaric rhizosphere; Eutric.BS: Cambisol Eutric bulk soil; Eutric.Rh: Cambisol Eutric rhizosphere; Hyperdystric.BS: Cambisol Hyperdystric bulk soil; Hyperdystric.Rh: Cambisol Hyperdystric rhizosphere.

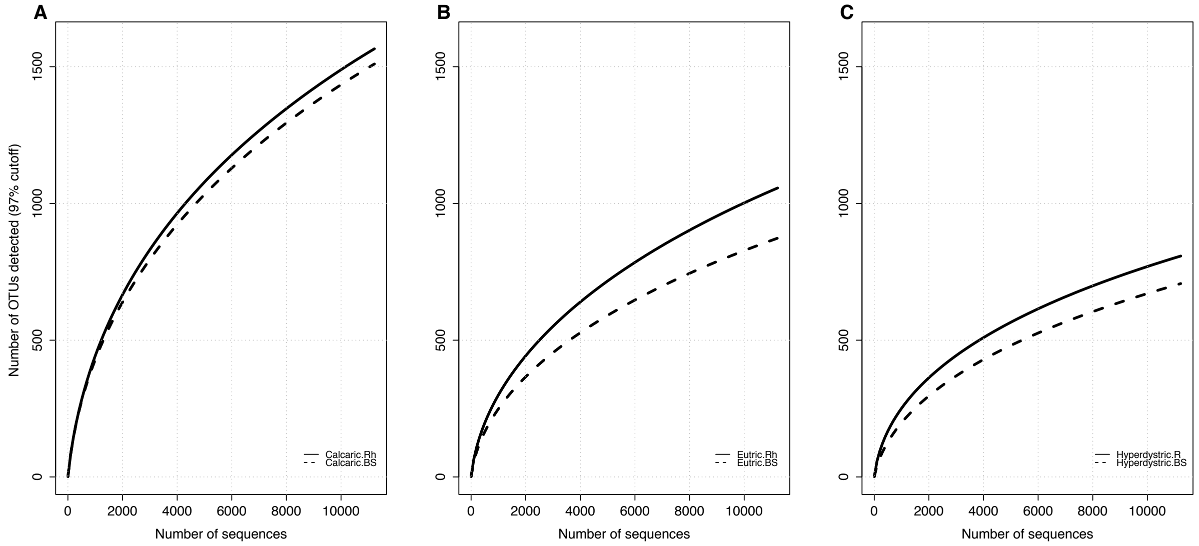

Figure S3. Shift in taxonomic diversity of bulk soil and rhizosphere-associated bacterial communities across the soil toposequence of Montiers and between bulk soil and rhizosphere compartments in each soil type. Multivariate analyses were performed at the OTU level and were conducted separately on bulk soil (BS, **Panel A**) and rhizosphere (Rh, **Panel B**) samples or on each soil types **(Calcaric samples, C; Eutric samples, D; Hyperdystric samples, E)** to determine a potential rhizosphere effect. The origins of samples are indicated as follows: filled orange squares, bulk soil samples from Calcaric; open orange squares, rhizosphere samples from Calcaric; filled green circles, bulk soil samples from Eutric, open green circles, rhizosphere samples from Eutric; filled blue triangles, bulk soil samples from Hyperdystric; open blue triangles, rhizosphere samples from Hyperdystric. Ellipses correspond to 95% confidence intervals about the mean.

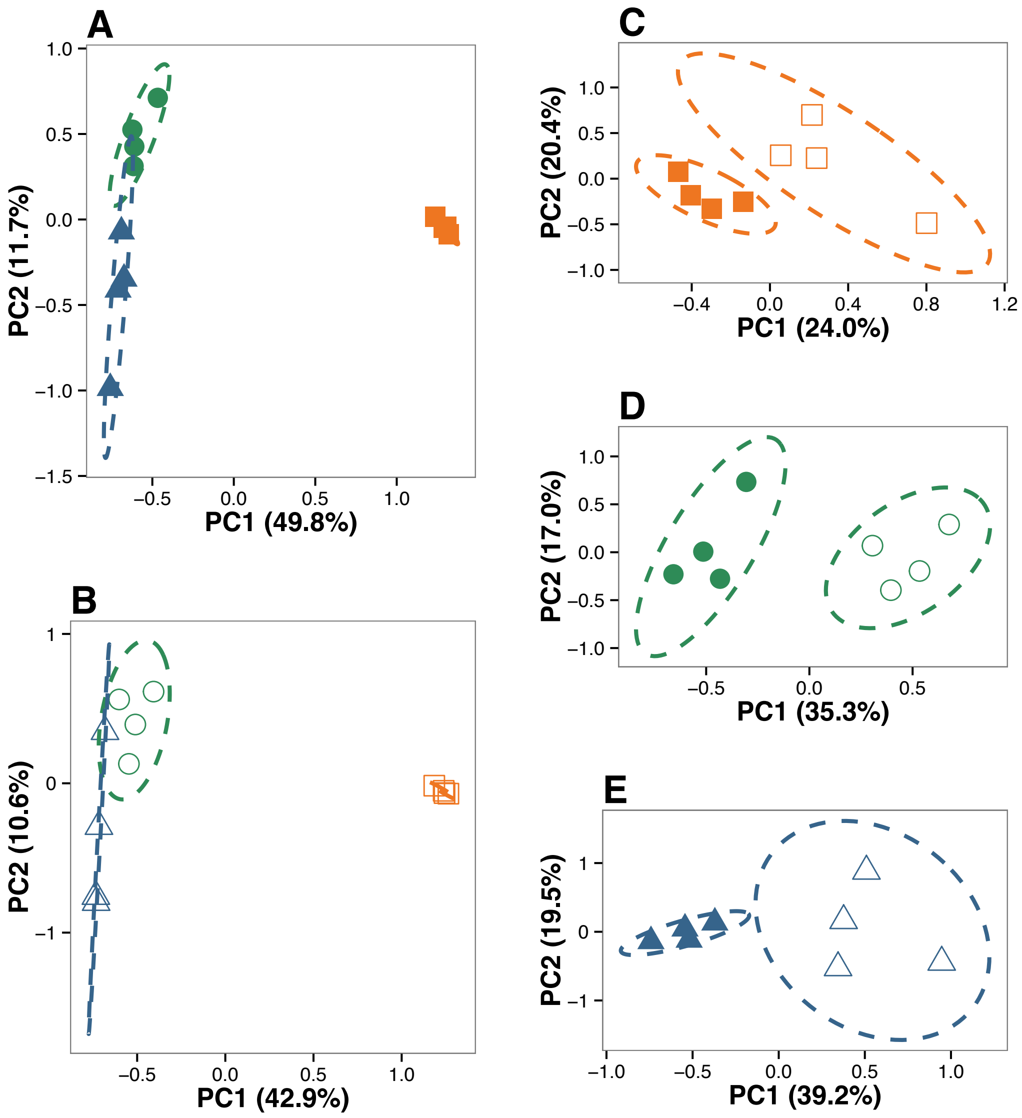

Figure.S4 Estimation of the number of functional genes shared: (A) among the bulk soil (BS) samples or the rhizosphere (Rh) samples across the toposequence and (B) between bulk soil and rhizosphere samples for each soil type.

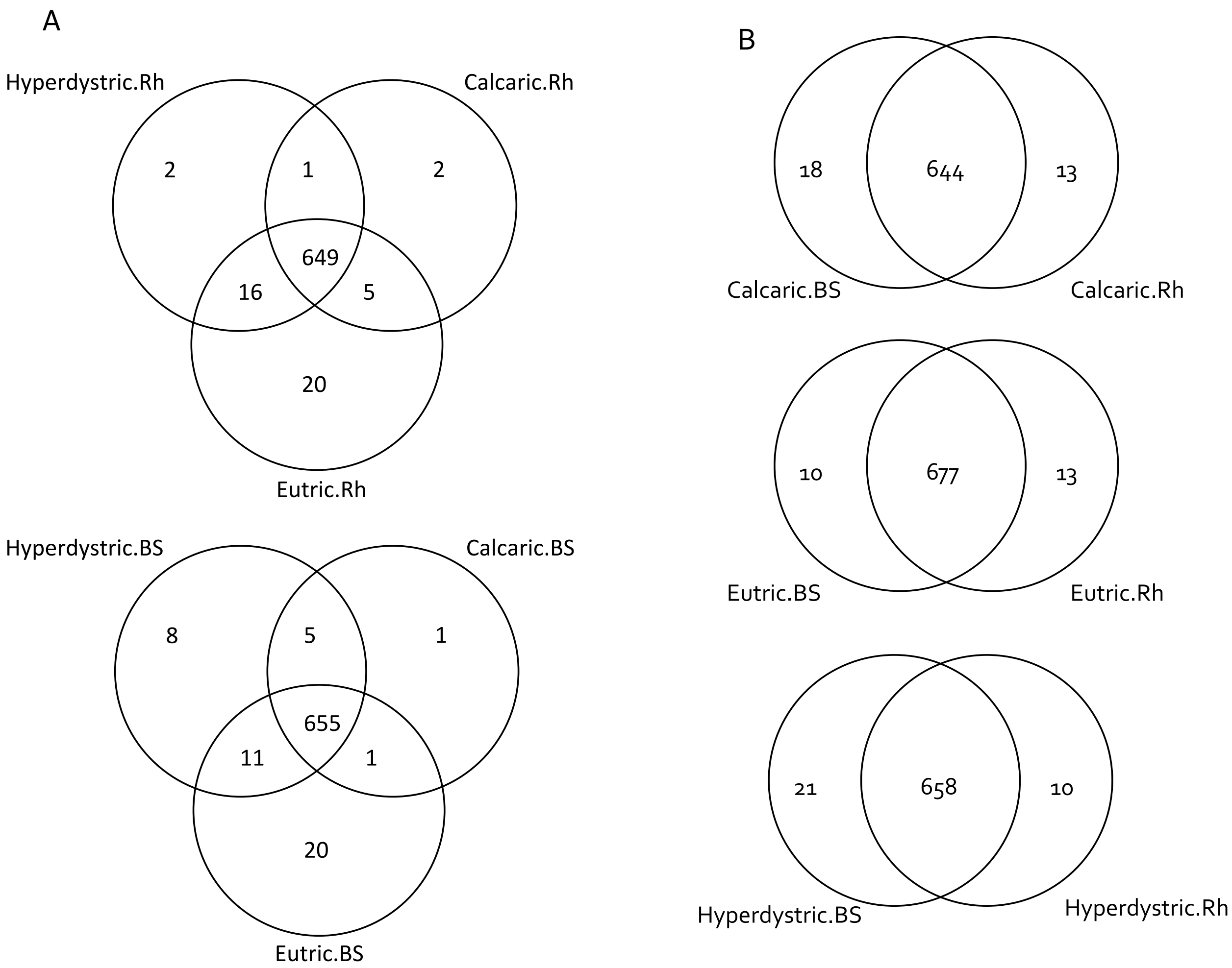

Figure.S5: Normalized signal intensity of probes detected from different gene categories. The signal intensities are the mean of detected individual probes averaged among the 3 biological replicates in the Cambisol Calcaric (A), Eutric (B) and Hyperdystric (C). All data are presented as mean (± Standard Error of the Mean). The significant differences among the bulk soil samples and their respective rhizosphere (P<0.05) are indicated above the bars (*).

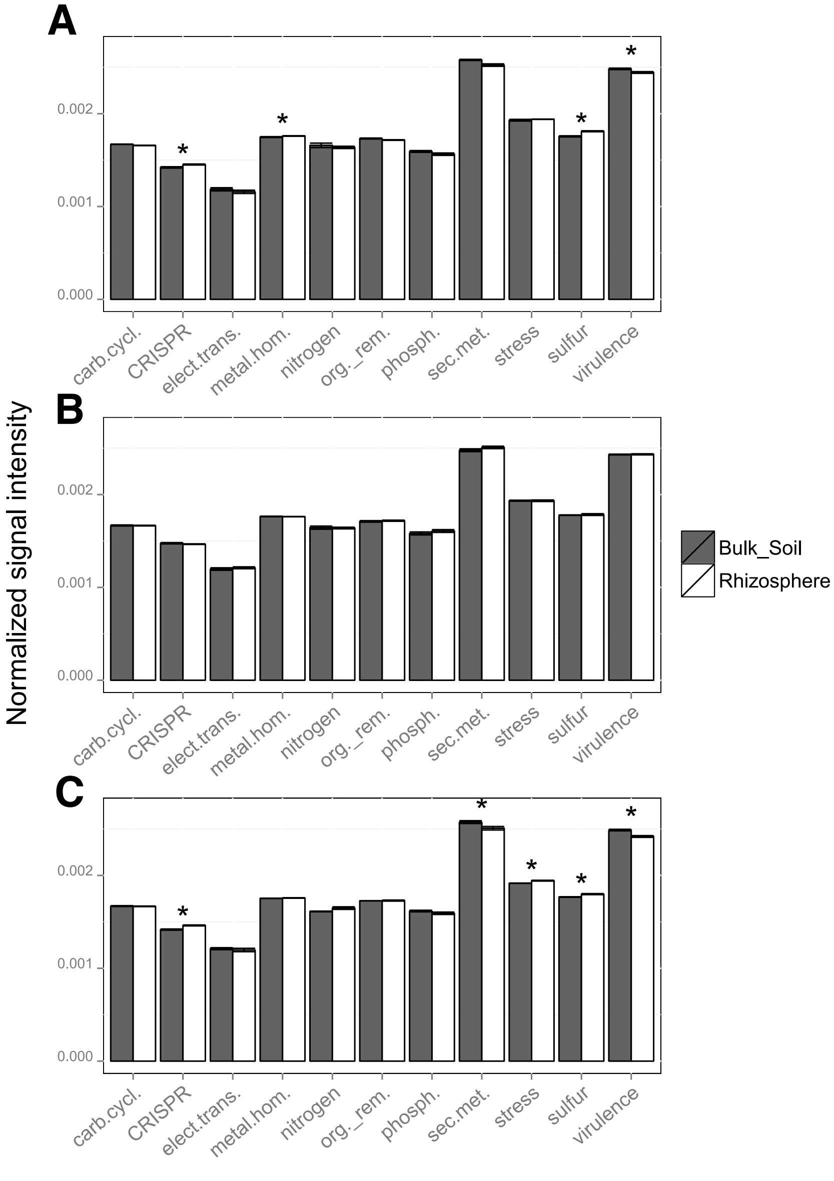

Figure.S6 Normalized signal intensity of genes involved in metal transport. The signal intensities are the mean of detected individual genes averaged among the 3 replicates from the Cambisol Calcaric (A), Eutric (B) and Hyperdystric (C). All data are presented as mean (± Standard Error of the Mean). The significant differences among the bulk soil (BS, grey) samples and their respective rhizosphere (R, white) are indicated by an asterisk above the bars ($p<0.05$).

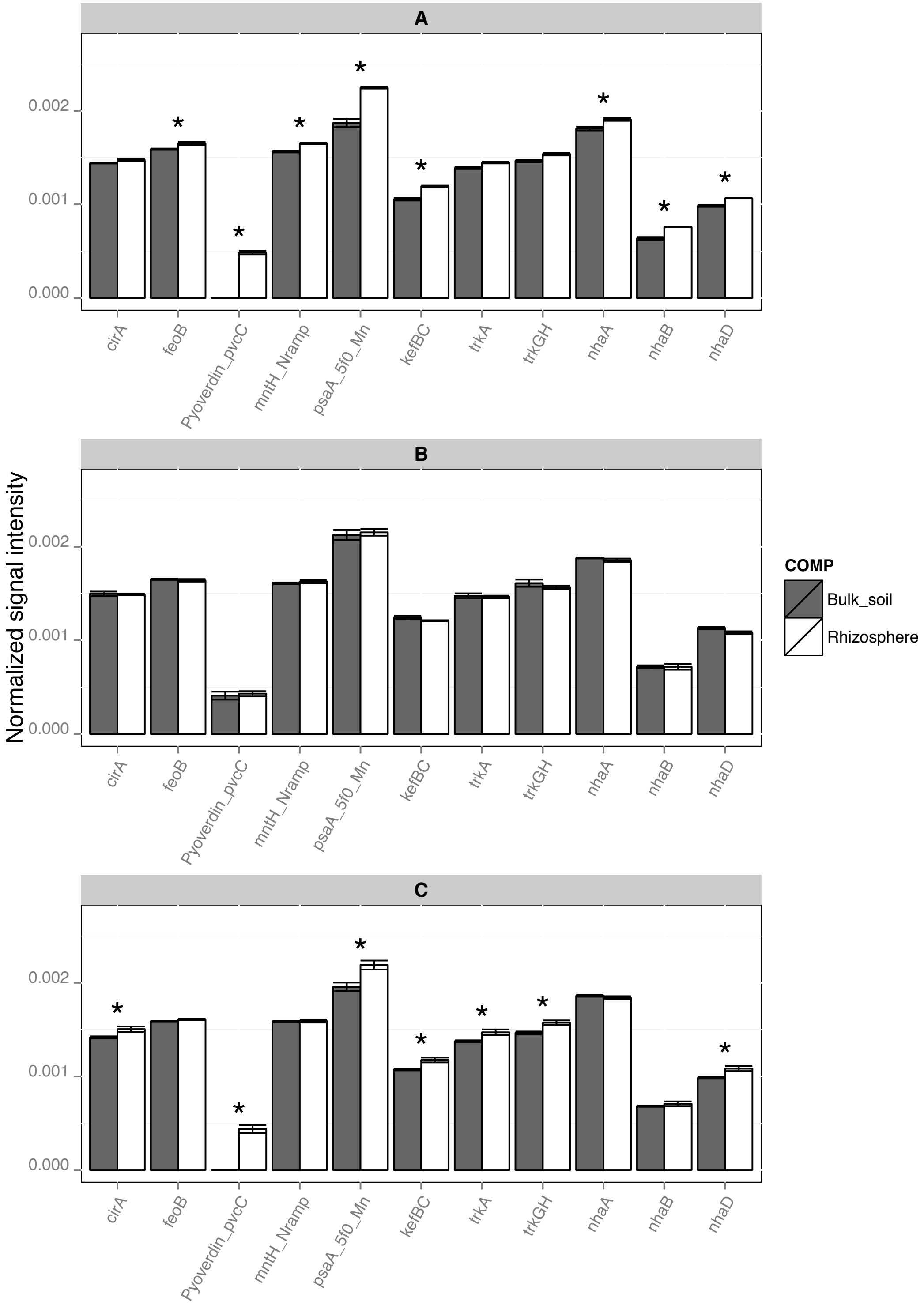

Figure.S7. Heatmap analysis of genes involved in nutrient cycling. The signal intensities are the mean of detected individual probes averaged among the 3 replicates for each compartment and soil type considered. Blue indicates signal intensities above background, whereas white indicates signal intensities below background. For each heatmap, the probe code (geochip code) and the taxonomic assignation are presented and the data are organized from the probes giving the higher signal at the top to the probes giving the lower signal at the bottom. For each function, only the top 10 is presented.

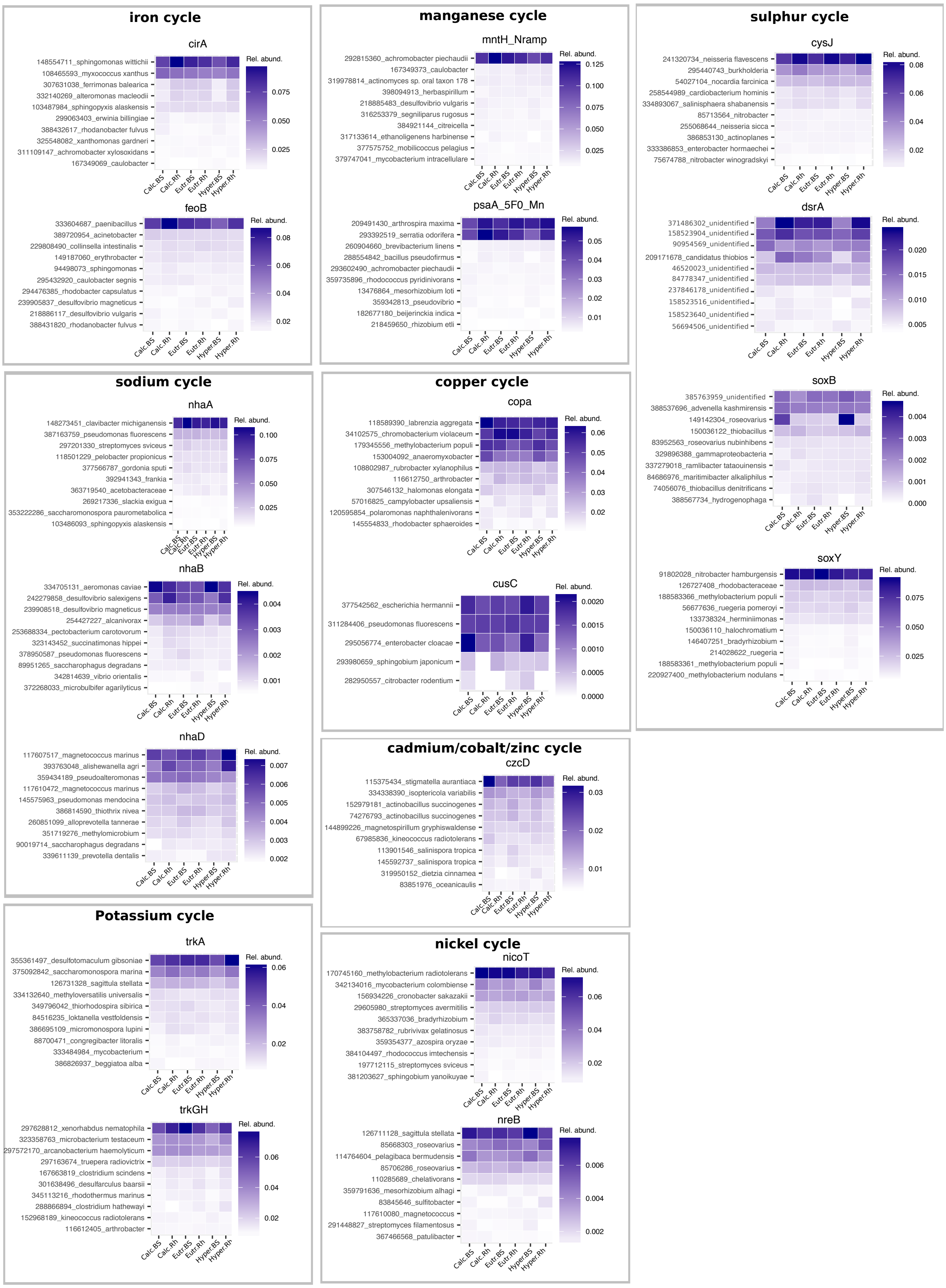

iron cycle
cirA
148554711_sphingomonas wittichii
108465593_myxococcus xanthus
307631038_ferrimonas balearica
332140269_alteromonas macleodii
103487984_sphingopyxis alaskensis
299063403_erwinia billingiae
388432617_rhodanobacter fulvus
325548082_xanthomonas gardneri
311109147_achromobacter xylosoxidans
167349069_caulobacter
Rel. abund.
0.075
0.050
0.025
feoB
333604687_paenibacillus
389720954_acinetobacter
229808490_collinsella intestinalis
149187060_erythrobacter
94498073_sphingomonas
295432920_caulobacter segnis
294476385_rhodobacter capsulatus
239905837_desulfovibrio magneticus
218886117_desulfovibrio vulgaris
388431620_rhodanobacter fulvus
Rel. abund.
0.08
0.06
0.04
0.02
manganese cycle
mntH_Nramp
292815360_achromobacter piechaudii
167349373_caulobacter
319978814_actinomyces sp. oral taxon 178
398094913_herbaspirillum
218885483_desulfovibrio vulgaris
316253379_segniliparus rugosus
384921144_citreicella
317133614_ethanoligenens harbinense
377575752_mobilicoccus pelagius
379747041_mycobacterium intracellulare
Rel. abund.
0.125
0.100
0.075
0.050
0.025
psaA_5F0_Mn
209491430_arthrospira maxima
293392519_serratia odorifera
260904660_brevibacterium linens
288554842_bacillus pseudofirmus
293602490_achromobacter piechaudii
359735896_rhodococcus pyridinivorans
13476864_mesorhizobium loti
359342813_pseudovibrio
182677180_beijerinckia indica
218459650_rhizobium etli
Rel. abund.
0.05
0.04
0.03
0.02
0.01
sulphur cycle
cysJ
241320734_neisseria flavescens
295440743_burkholderia
54027104_nocardia farcinica
258544989_cardiobacterium hominis
334893067_salinisphaera shabanensis
85713564_nitrobacter
255068644_neisseria sicca
386853130_actinoplanes
333386853_enterobacter hormaechei
75674788_nitrobacter winogradskyi
Rel. abund.
0.08
0.06
0.04
0.02
dsrA
371486302_unidentified
158523904_unidentified
90954569_unidentified
209171678_candidatus thiobios
46520023_unidentified
84778347_unidentified
237846178_unidentified
158523516_unidentified
158523640_unidentified
56694506_unidentified
Rel. abund.
0.020
0.015
0.010
0.005
soxB
385763959_unidentified
388537696_advenella kashmirensis
149142304_roseovarius
150036122_thiobacillus
83952563_roseovarius nubinhibens
329896388_gammaproteobacteria
337279018_ramlibacter tataouinensis
84686976_maritimibacter alkaliphilus
74056076_thiobacillus denitrificans
388567734_hydrogenophaga
Rel. abund.
0.004
0.003
0.002
0.001
0.000
soxY
91802028_nitrobacter hamburgensis
126727408_rhodobacteraceae
188583366_methylobacterium populi
56677636_ruegeria pomeroyi
133738324_herminiimonas
150036110_halochromatium
146407251_bradyrhizobium
214028622_ruegeria
188583361_methylobacterium populi
220927400_methylobacterium nodulans
Rel. abund.
0.075
0.050
0.025
sodium cycle
nhaA
148273451_clavibacter michiganensis
387163759_pseudomonas fluorescens
297201330_streptomyces sviceus
118501229_pelobacter propionicus
377566787_gordonia sputi
392941343_frankia
363719540_acetobacteraceae
269217336_slackia exigua
353222286_saccharomonospora paurometabolica
103486093_sphingopyxis alaskensis
Rel. abund.
0.100
0.075
0.050
0.025
nhaB
334705131_aeromonas caviae
242279858_desulfovibrio salexigens
239908518_desulfovibrio magneticus
254427227_alcanivorax
253688334_pectobacterium carotovorum
323143452_succinatimonas hippei
378950587_pseudomonas fluorescens
89951265_saccharophagus degradans
342814639_vibrio orientalis
372268033_microbulbifer agarilyticus
Rel. abund.
0.004
0.003
0.002
0.001
nhaD
117607517_magnetococcus marinus
393763048_alishewanella agri
359434189_pseudoalteromonas
117610472_magnetococcus marinus
145575963_pseudomonas mendocina
386814590_thiothrix nivea
260851099_alloprevotella tannerae
351719276_methylomicrobium
90019714_saccharophagus degradans
339611139_prevotella dentalis
Rel. abund.
0.007
0.006
0.005
0.004
0.003
0.002
copper cycle
copa
118589390_labrenzia aggregata
34102575_chromobacterium violaceum
179345556_methylobacterium populi
153004092_anaeromyxobacter
108802987_rubrobacter xylanophilus
116612750_arthrobacter
307546132_halomonas elongata
57016825_campylobacter upsaliensis
120595854_polaromonas naphthalenivorans
145554833_rhodobacter sphaeroides
Rel. abund.
0.06
0.05
0.04
0.03
0.02
cusC
377542562_escherichia hermannii
311284406_pseudomonas fluorescens
295056774_enterobacter cloacae
293980659_sphingobium japonicum
282950557_citrobacter rodentium
Rel. abund.
0.0020
0.0015
0.0010
0.0005
0.0000
cadmium/cobalt/zinc cycle
czcD
115375434_stigmatella aurantiaca
334338390_isoptericola variabilis
152979181_actinobacillus succinogenes
74276793_actinobacillus succinogenes
144899226_magnetospirillum gryphiswaldense
67985836_kineococcus radiotolerans
113901546_salinispora tropica
145592737_salinispora tropica
319950152_dietzia cinnamea
83851976_oceanicaulis
Rel. abund.
0.03
0.02
0.01
Potassium cycle
trkA
355361497_desulfotomaculum gibsoniae
375092842_saccharomonospora marina
126731328_sagittula stellata
334132640_methyloversatilis universalis
349796042_thiorhodospira sibirica
84516235_loktanella vestfoldensis
386695109_micromonospora lupini
88700471_congregibacter litoralis
333484984_mycobacterium
386826937_beggiatoa alba
Rel. abund.
0.06
0.04
0.02
trkGH
297628812_xenorhabdus nematophila
323358763_microbacterium testaceum
297572170_arcanobacterium haemolyticum
297163674_truepera radiovictrix
167663819_clostridium scindens
301638496_desulfarculus baarsii
345113216_rhodothermus marinus
288866894_clostridium hathewayi
152968189_kineococcus radiotolerans
116612405_arthrobacter
Rel. abund.
0.06
0.04
0.02
nickel cycle
nicoT
170745160_methylobacterium radiotolerans
342134016_mycobacterium colombiense
156934226_cronobacter sakazakii
29605980_streptomyces avermitilis
365337036_bradyrhizobium
383758782_rubrivivax gelatinosus
359354377_azospira oryzae
384104497_rhodococcus imtechensis
197712115_streptomyces sviceus
381203627_sphingobium yanoikuyae
Rel. abund.
0.06
0.04
0.02
nreB
126711128_sagittula stellata
85668303_roseovarius
114764604_pelagibaca bermudensis
85706286_roseovarius
110285689_chelativorans
359791636_mesorhizobium alhagi
83845646_sulfitobacter
117610080_magnetococcus
291448827_streptomyces filamentosus
367466568_patulibacter
Rel. abund.
0.006
0.004
0.002
Calc.BS
Calc.Rh
Eutr.BS
Eutr.Rh
Hyper.BS
Hyper.Rh

Figure.S8 Normalized signal intensity of genes involved in the CRISPR system. The signal intensities are the mean of detected individual genes averaged among the 3 replicates from the Cambisol Calcaric (A), Eutric (B) and Hyperdystric (C). All data are presented as mean (± Standard Error of the Mean). The significant differences among the bulk soil (BS, grey) samples and their respective rhizosphere (R, white) are indicated by an asterisk above the bars (p<0.05).

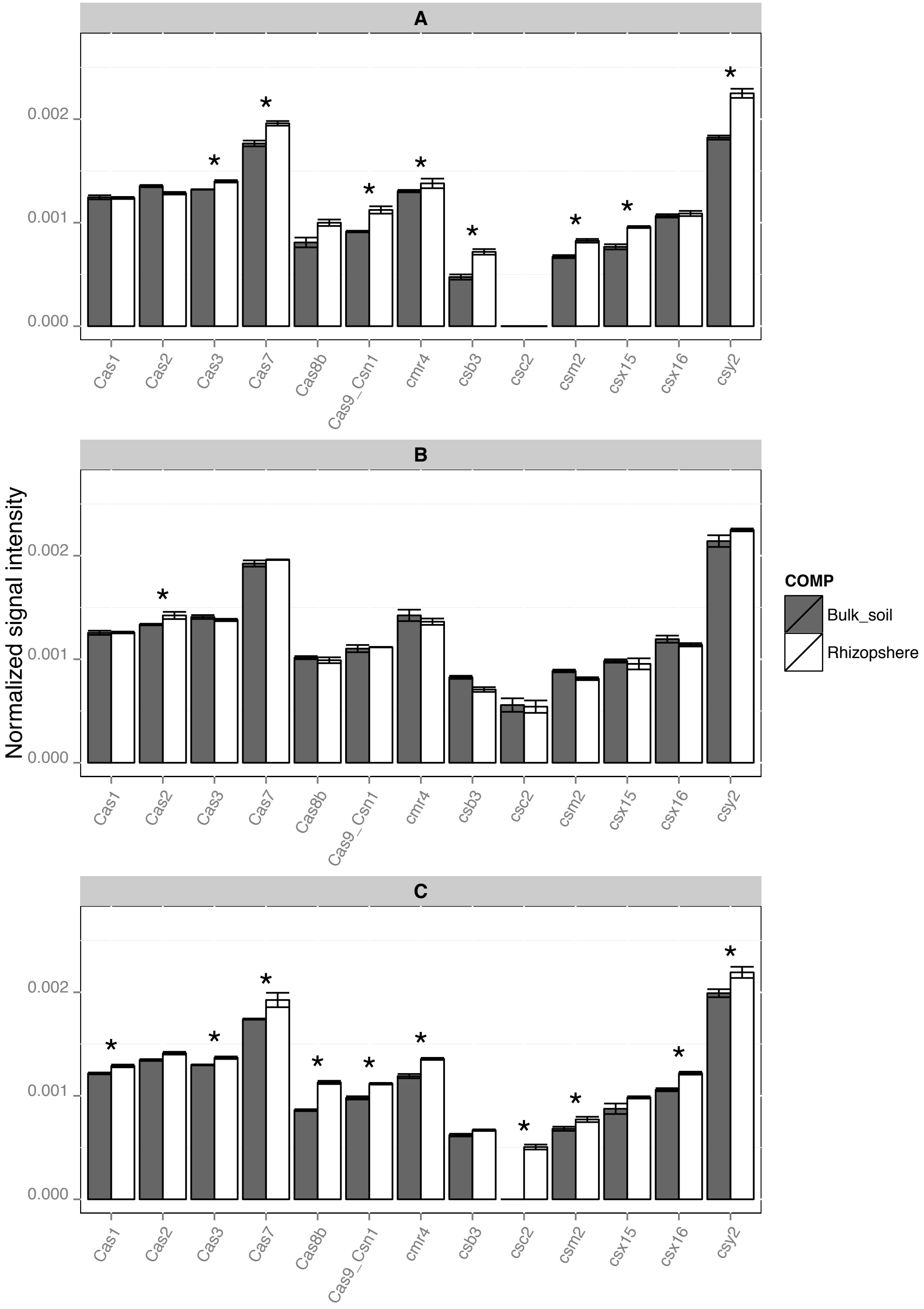

Figure.S9. Heatmap analysis of genes involved in microbial interactions. The signal intensities are the mean of detected individual probes averaged among the 3 replicates for each compartment and soil type considered. Blue indicates signal intensities above background, whereas white indicates signal intensities below background. For each heatmap, the probe code (geochip code) and the taxonomic assignation are presented and the data are organized from the probes giving the higher signal at the top to the probes giving the lower signal at the bottom. For each function, only the top 10 is presented.

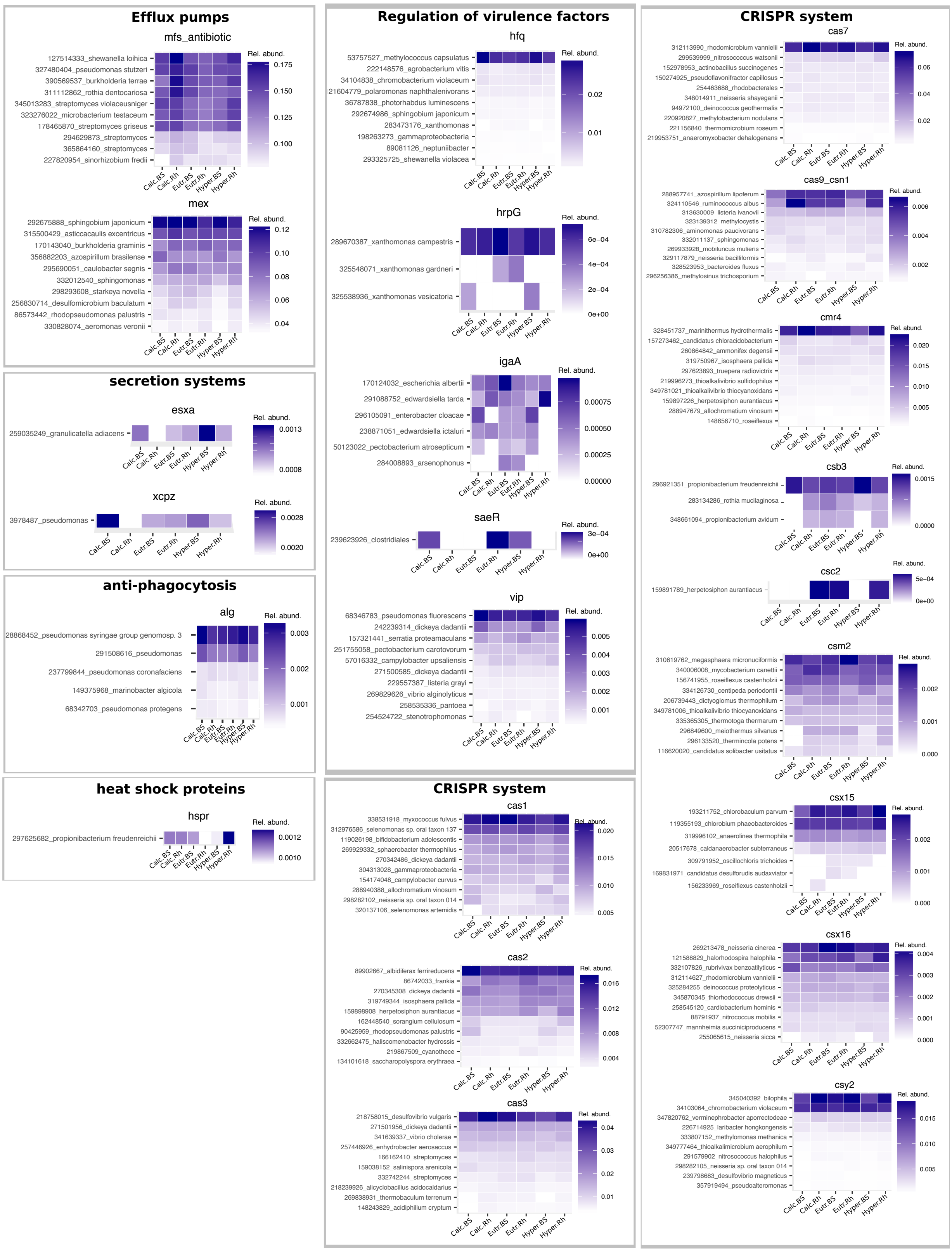
Efflux pumps
mfs_antibiotic
mex
secretion systems
esxa
xcpz
anti-phagocytosis
alg
heat shock proteins
hspr
Regulation of virulence factors
hfq
hrpG
igaA
saeR
vip
CRISPR system
cas1
cas2
cas3
CRISPR system
cas7
cas9_csn1
cmr4
csb3
csc2
csm2
csx15
csx16
csy2
Rel. abund.

Figure.S10 Normalized signal intensity of genes involved in carbon degradation. The signal intensities are the mean of detected individual genes averaged among the 3 replicates from the Calcaric (A), Eutric (B) and Hyperdystric (C). All data are presented as mean (± Standard Error of the Mean). The significant differences among the bulk soil (BS, grey) samples and their respective rhizosphere (R, white) are indicated by an asterisk above the bars (p<0.05).

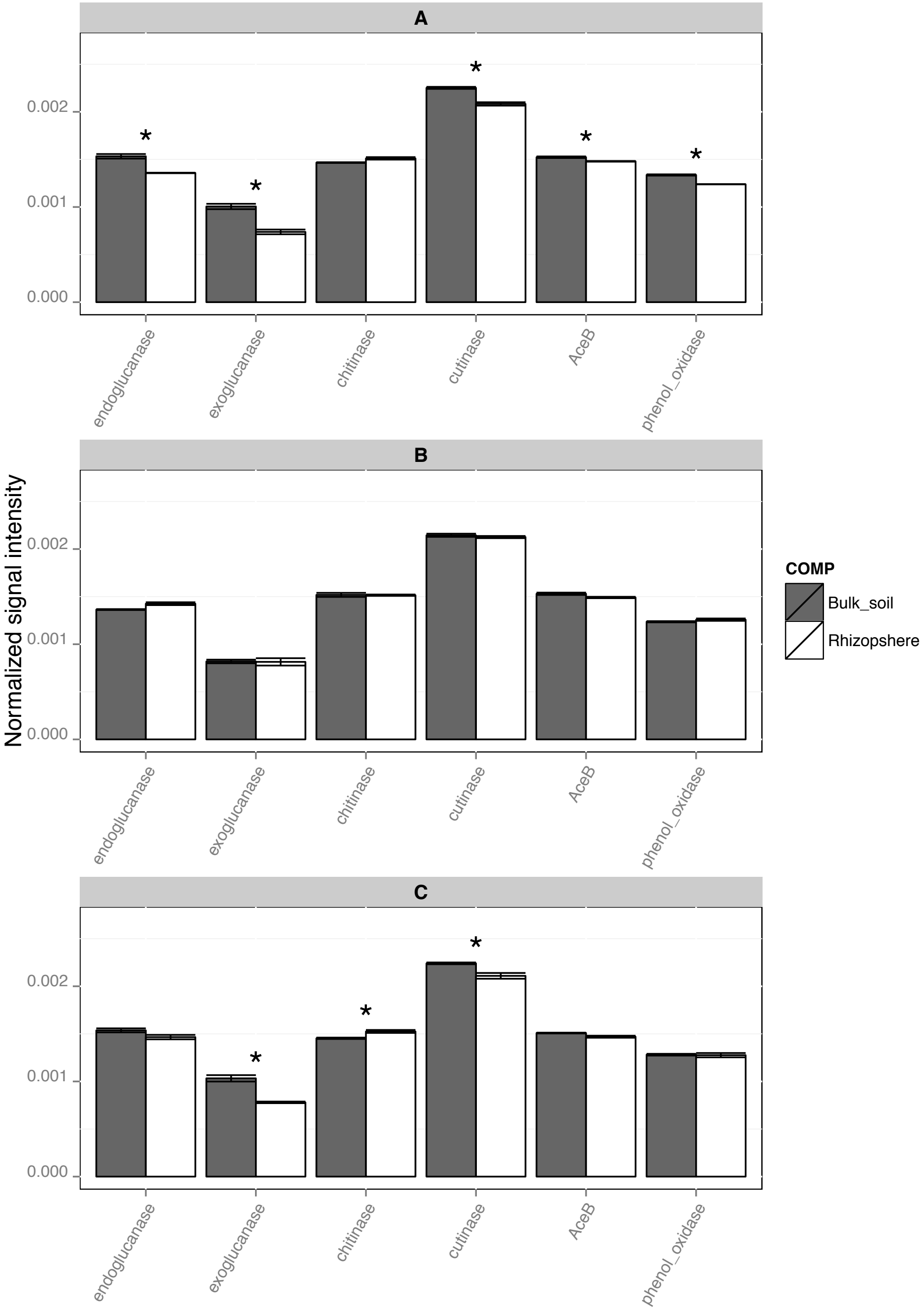

Figure.S11. Heatmap analysis of genes involved in carbon cycling. The signal intensities are the mean of detected individual probes averaged among the 3 replicates for each compartment and soil type considered. Blue indicates signal intensities above background, whereas white indicates signal intensities below background. For each heatmap, the probe code (geochip code) and the taxonomic assignation are presented and the data are organized from the probes giving the higher signal at the top to the probes giving the lower signal at the bottom. For each function, only the top 10 is presented.

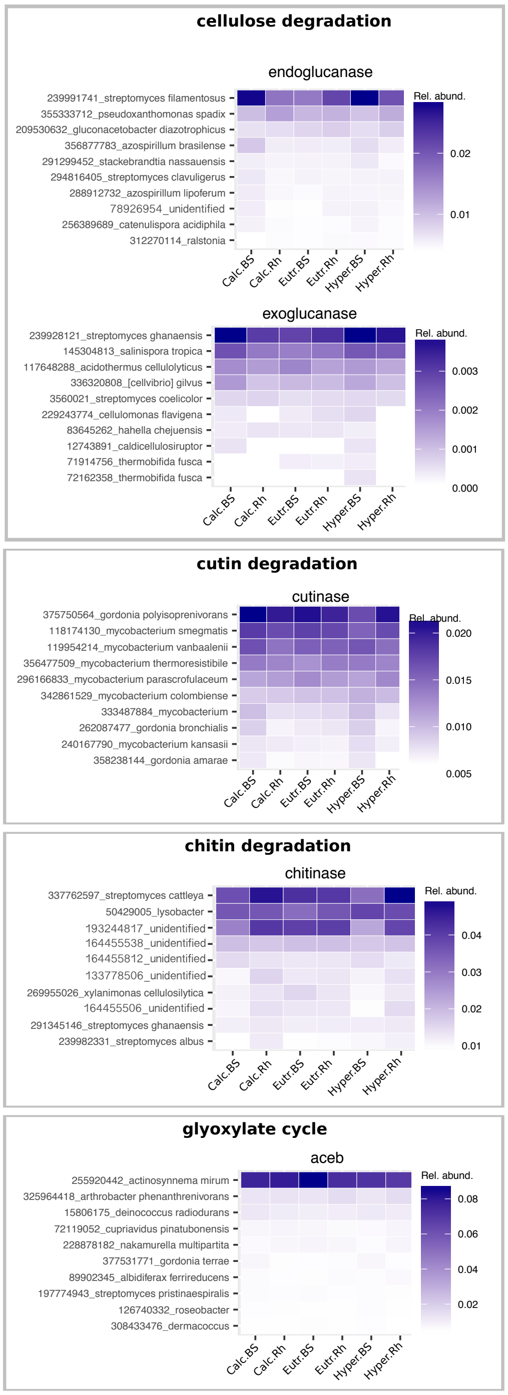

Figure S12: Partitioning of the variance in soil bacterial communities by explanatory variables (soil type and compartment). Barplots represent the variance explained by the soil variables effect, the compartment effect as well as the unexplained variance for each data measured (16S rRNA pyrosequences, Geochip functional categories and Biolog data).

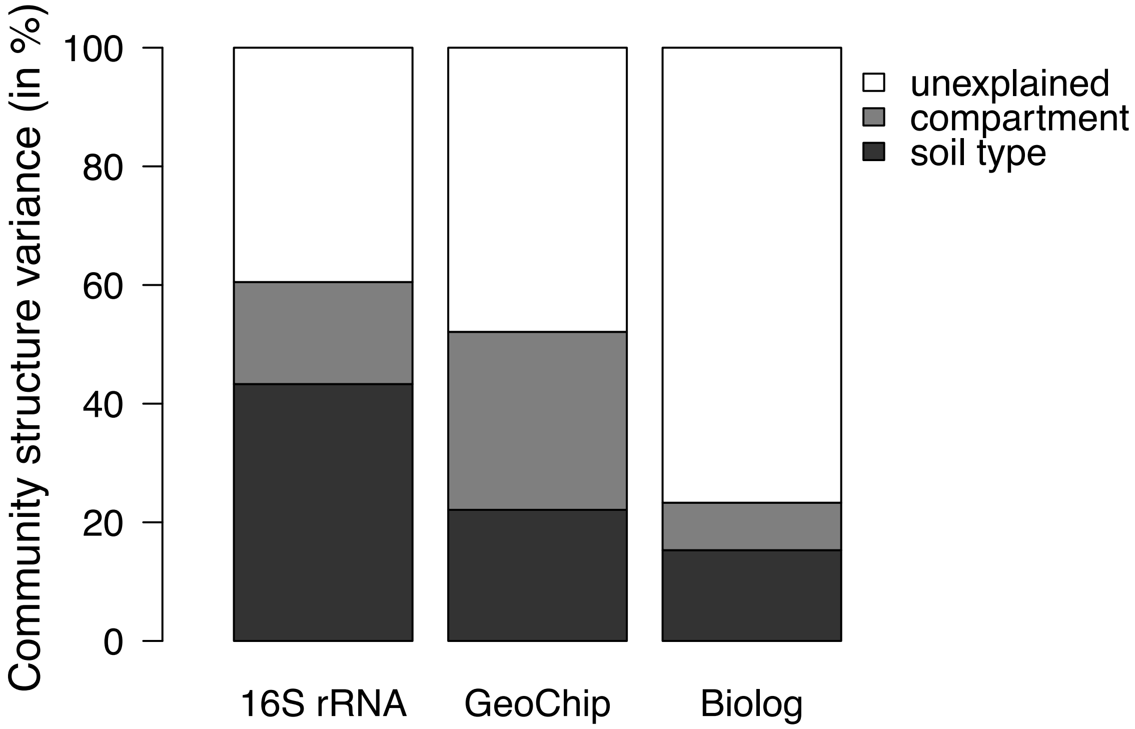

Table S1: Physico-chemical characteristics of the soil samples collected along the toposequence of Montiers. Soil analyses have been done at the Soil analysis platform of Arras (INRA, France) according to the protocols described in the material and methods section. Values are averages of 4 replicates (n=4). Several parameters measured have been abbreviated in the table as follows: Water cont., Water content; N, Total, total nitrogen; C tot, Total carbon; CEC, Cationic exchange capacity; P. Duch, Phosphorus extracted according to the Duchaufour method; P. Ols, Phosphorus extracted according to the Olsen method; OM, Organic matter; Limest., Limestone; H+, Exchangeable protons. For each soil parameter, significant differences are presented by different letters (a,b,c; P<0.05)

Soil type	pH	Water cont. (in %)	N tot (g/kg)	C tot (g/kg)	C/N	P.Duch (g/kg)	P.Ols (g/kg)	OM (g/kg)	Limest. (g/kg)
**Calcaric**	7.14^{a}± 0.03	39.65^{a}± 1.55	4.89^{a}± 0.40	66.88^{a}± 6.25	13.65^{a}± 0.19	0.34^{a}± 0.03	0.04^{a}± 0.00	115.63^{a}± 10.81	7.25± 1.03
**Eutric**	5.01^{b}± 0.04	16.83^{b}± 0.77	1.46^{b}± 0.09	21.15^{b}± 0.54	14.63^{a}± 0.52	0.22^{a}± 0.02	0.01^{a}± 0.00	36.55^{b}± 0.93	<1
**Hyperdystric**	4.63^{c}± 0.04	11.50^{c}± 0.33	1.19^{b}± 0.07	17.20^{b}± 0.74	14.53^{a}± 0.53	0.38^{a}± 0.13	0.03^{a}± 0.02	29.78^{b}± 1.30	<1

Soil type	CEC (cmol+/kg)	H+	Mg	Ca	K	Na	Mn	Al	Fe
**Calcaric**	48.30^{a}± 2.04	<0.05	0.81^{a}± 0.14	48.80^{a}± 2.03	0.29^{a}± 0.01	0.07^{a}± 0.00	0.02^{c}± 0.00	0.11^{c}± 0.01	0.02^{a}± 0.00
**Eutric**	6.45^{b}± 0.59	0.32^{a}± 0.01	0.25^{b}± 0.04	2.51^{b}± 0.61	0.10^{b}± 0.01	0.04^{b}± 0.00	0.12^{b}± 0.03	3.06^{a}± 0.22	0.03^{a}± 0.00
**Hyperdystric**	2.66^{b}± 0.16	0.33^{a}± 0.01	0.08^{b}± 0.01	0.26^{b}± 0.05	0.07^{b}± 0.01	0.01^{c}± 0.00	0.23^{a}± 0.03	2.17^{b}± 0.10	0.02^{a}± 0.00

Table S2: Estimates of the taxonomic (16S rRNA gene libraries), functional (GeoChip microarray and Biolog) bacterial diversity for bulk soil and rhizosphere samples along the toposequence of Montiers. Each value is the mean of biological replicates (± Standard Error of the Mean). For each indicator, significant differences are presented by different letters (P<0.05).

**Samples**	**Compartment**	**16S rRNA gene libraries**				**GeoChip microarray**		**Biolog Ecoplate**	
		**No. of OTUs (0.03%)**	**Shannon index**	**Chao1 index**	**Coverage**	**No. of genes**	**Shannon index**	**No. of substrates metabolized**	**Shannon index**
**Calcaric**	**Bulk Soil**	1510^{a} ± 10	6.12^{a} ± 0.02	2444^{a} ± 19	0.94 ± 0.001	655^{b} ± 7.3	6.48^{b} ± 0.01	28.50^{a} ± 2.38	3.17^{a} ± 0.13
**Calcaric**	**Rhizosphere**	1566^{a} ± 44	6.16^{a} ± 0.07	2478^{a} ± 57	0.94 ± 0.001	654^{b} ± 0.3	6.48^{b} ± 0.00	28.50^{a} ± 1.29	3.18^{a} ± 0.03
**Eutric**	**Bulk Soil**	881^{b} ± 65	4.44^{cd} ± 0.24	1523^{bc} ± 121	0.96 ± 0.003	681^{a} ± 1.0	6.52^{a} ± 0.00	29.25^{a} ± 1.50	3.02^{ab} ± 0.09
**Eutric**	**Rhizosphere**	1057^{b} ± 66	5.18^{b} ± 0.12	1767^{b} ± 69	0.96 ± 0.002	683^{a} ± 6.8	6.53^{a} ± 0.01	24.25 ^{a}b ± 4.57	2.73^{c} ± 0.21
**Hyperdystric**	**Bulk Soil**	707^{c} ± 59	3.78^{d} ± 0.25	1197^{c} ± 113	0.97 ± 0.003	674^{ab} ± 3.5	6.51^{ab} ± 0.01	27.00^{ab} ± 1.82	2.87^{bc} ± 0.06
**Hyperdystric**	**Rhizosphere**	807^{c} ± 23	4.76^{bc} ± 0.18	1299^{c} ± 72	0.97 ± 0.002	662^{ab} ± 2.2	6.50^{ab} ± 0.00	22.75^{b} ± 1.71	2.69^{c} ± 0.07

Table.S3: Changes in taxonomic composition along the toposequence for bulk soil and rhizosphere samples. Text in bold indicates significant differences. Samples are referred as follow: CC: Calcaric Cambisol; EC: Eutric Cambisol; HC: Hyperdystric Cambisol. The symbols ‘>’ and ‘<’ mean significantly more or less abundant and ‘=’ not significantly different.

BULK_SOIL SAMPLES			RHIZOPHERE SAMPLES		
PHYLUM	CLASS	ORDER	PHYLUM	CLASS	ORDER
**Acidobacteria HC>EC>CC**	**Acidobacteria_Gp1 CC<EC>HC**	**Acidobacteria_Gp1 CC<EC>HC**	**Acidobacteria HC=EC>CC**	**Acidobacteria_Gp1 HC=EC>CC**	**Acidobacteria_Gp1 HC=EC>CC**
	**Acidobacteria_Gp2 CC<EC>HC**	**Acidobacteria_Gp2 CC<EC>HC**		**Acidobacteria_Gp2 HC=EC>CC**	**Acidobacteria_Gp2 HC=EC>CC**
	**Acidobacteria_Gp3 EC>CC**	**Acidobacteria_Gp3 EC>CC**		**Acidobacteria_Gp3 HC=EC>CC**	**Acidobacteria_Gp3 HC=EC>CC**
	Acidobacteria_Gp4	Acidobacteria_Gp4		Acidobacteria_Gp4	Acidobacteria_Gp4
	**Acidobacteria_Gp5 CC>EC=HC**	**Acidobacteria_Gp5 CC>EC=HC**		**Acidobacteria_Gp5 CC>EC=HC**	**Acidobacteria_Gp5 CC>EC=HC**
	Acidobacteria_Gp6	Acidobacteria_Gp6		**Acidobacteria_Gp6 CC>EC=HC**	**Acidobacteria_Gp6 CC>EC=HC**
	Acidobacteria_Gp7	Acidobacteria_Gp7		**Acidobacteria_Gp7 CC=EC>HC**	**Acidobacteria_Gp7 CC=EC>HC**
	**Acidobacteria_Gp11 CC>EC=HC**	**Acidobacteria_Gp11 CC>EC=HC**		**Acidobacteria_Gp11 CC>EC=HC**	**Acidobacteria_Gp11 CC>EC=HC**
	**Acidobacteria_Gp22 CC>EC=HC**	**Acidobacteria_Gp22 CC>EC=HC**		**Acidobacteria_Gp22 CC>EC=HC**	**Acidobacteria_Gp22 CC>EC=HC**
	**Acidobacteria_Gp25 CC>EC=HC**	**Acidobacteria_Gp25 CC>EC=HC**		Acidobacteria_Gp25 ND	Acidobacteria_Gp25 ND
	Holophagae	Holophagales		Holophagae	Holophagales
	**Unclassified CC>EC=HC**	**Unclassified CC>EC=HC**		**Unclassified CC>EC**	**Unclassified CC>EC**
**Actinobacteria CC>EC=HC**	**Actinobacteria CC>EC=HC**	**Acidimicrobiales CC>EC=HC**	Actinobacteria	Actinobacteria	**Acidimicrobiales CC>HC**
		**Actinomycetales CC>HC**			Actinomycetales
		Bifidobacteriales			Bifidobacteriales ND
		**Solirubrobacterales CC>EC=HC**			**Solirubrobacterales CC>EC=HC**
		**unclassified CC>EC=HC**			**unclassified CC>HC**
**Bacteroidetes CC>EC=HC**	**Flavobacteria CC>EC=HC**	**Flavobacteriales CC>EC=HC**	**Bacteroidetes CC>EC=HC**	Flavobacteria	Flavobacteriales
	**Sphingobacteria CC>EC=HC**	**Sphingobacteriales CC>EC=HC**		**Sphingobacteria CC>EC**	Sphingobacteriales CC>EC
	**unclassified CC>EC=HC**	**unclassified CC>EC=HC**		**unclassified CC>EC=HC**	**unclassified CC>EC=HC**
Chlamydiae	Chlamydiae	Chlamydiales	Chlamydiae	Chlamydiae	Chlamydiales
**Chloroflexi CC>EC=HC**	**Chloroflexi CC>EC=HC**	**Chloroflexi CC>EC=HC**	**Chloroflexi CC>EC=HC**	**Chloroflexi CC>EC=HC**	**Chloroflexi CC>EC=HC**
	**Unclassified CC>EC=HC**	**Unclassified CC>EC=HC**		**Unclassified CC>EC=HC**	**Unclassified CC>EC=HC**
Deinococcus	Deinococcus	Deinococcus	Deinococcus ND	Deinococcus ND	Deinococcales ND
**Firmicutes CC>EC=HC**		**Bacillales CC>EC=HC**	**Firmicutes CC>EC=HC**		**Bacillales CC>EC=HC**
	**Bacilli CC>EC=HC**	Lactobacillales		**Bacilli CC>EC=HC**	Lactobacillales
		unclassified ND			unclassified
	Clostridia	Clostridiales		Clostridia	Clostridiales
	Unclassified	Unclassified		unclassified	unclassified
**Gemmatimodetes CC>HC**	**Gemmatimodetes CC>HC**	**Gemmatimodales CC>HC**	**Gemmatimodetes CC>EC=HC**	**Gemmatimodetes CC>EC=HC**	**Gemmatimodales CC>EC=HC**
**Nitrospira CC>EC=HC**	**Nitrospira CC>EC=HC**	**Nitrospirales CC>EC=HC**	**Nitrospira CC>EC=HC**	**Nitrospira CC>EC=HC**	**Nitrospirales CC>EC=HC**
OP10	OP10	OP10	**OP10 EC>CC**	**OP11 EC>CC**	**OP11 EC>CC**
Planctomycetes	Planctomycetes	Planctomycetales	Planctomycetes	Planctomycetes	Planctomycetales
**Proteobacteria CC>EC=HC**	**Alphaproteobacteria CC>HC**	Caulobacterales	Proteobacteria	Alphaproteobacteria	Caulobacterales
		**Rhizobiales CC>EC=HC**			Rhizobiales
		**Rhodobacterales CC>EC=HC**			**Rhodobacterales CC>EC=HC**
		**Rhodospirillales EC>CC**			**Rhodospirillales HC=EC>CC**
		Rickettsiales			Rickettsiales
		**Sphingomodales CC>EC=HC**			**Sphingomodales CC>EC=HC**
		**unclassified CC>EC=HC**			**unclassified CC>EC=HC**
	**Betaproteobacteria CC>EC=HC**	**Burkholderiales CC>EC=HC**		**Betaproteobacteria CC>EC=HC**	Burkholderiales
		**Methylophilales CC>EC=HC**			Methylophilales
		Neisseriales ND			Neisseriales
		**Nitrosomodales CC>EC**			**Nitrosomodales CC>EC=HC**
		**Rhodocyclales CC>EC**			**Rhodocyclales CC>EC=HC**
		**unclassified CC>EC=HC**			**unclassified CC>EC=HC**
	**Deltaproteobacteria CC>EC=HC**	**Bdellovibrionales CC>EC=HC**		**Deltaproteobacteria CC>EC=HC**	**Bdellovibrionales CC>EC=HC**
		Desulfobacterales ND			Desulfobacterales
		**Desulfuromodales CC>EC=HC**			Desulfuromodales
		**Myxococcales CC>EC=HC**			**Myxococcales CC>EC=HC**
		**unclassified CC>EC=HC**			**unclassified CC>EC=HC**
	**Gammaproteobacteria CC>HC**	Aeromodales		Gammaproteobacteria	Aeromodales
		Alteromodales ND			Alteromodales
		Chromatiales			Chromatiales ND
		Enterobacteriales			Enterobacteriales
		Pseudomodales			Pseudomodales
		**unclassified CC>EC=HC**			**unclassified CC>EC=HC**
		Xanthomodales			Xanthomodales
	**Unclassified CC>EC=HC**	**Unclassified CC>EC=HC**		**unclassified CC>EC=HC**	**unclassified CC>EC=HC**
Spirochaetes ND	Spirochaetes ND	Spirochaetales ND	**Spirochaetes CC>EC=HC**	**Spirochaetes CC>EC=HC**	**Spirochaetales CC>EC=HC**
TM7	TM7	TM7	TM7	TM7	TM7
**Verrucomicrobia CC=EC>HC**	**Opitutae ND**	**Opitutales ND**	**Verrucomicrobia HC>CC**	Opitutae	Opitutales
	**Spartobacteria CC>EC=HC**	**Spartobacteria CC>EC=HC**		Spartobacteria	Spartobacteria
	**Subdivision3 CC>HC**	**Subdivision3 CC>HC**		Subdivision3	Subdivision3
	**unclassified CC>EC=HC**	**unclassified CC>EC=HC**		unclassified	unclassified
**WS3 CC>EC=HC**	**WS3 CC>EC=HC**	**unclassified CC>EC=HC**	WS3	WS3	unclassified

Table.S4 Part 1: Changes in taxonomic composition between bulk soil and rhizosphere samples for each soil type. Text in red and blue indicates a significant increase in rhizosphere and bulk soils samples, respectively. Samples are referred as follow: BS: Bulk Soil; Rh: Rhizosphere. The symbols '>' and '<' mean significantly more or less abundant and '=' not significantly different.

CALCARIC CAMBISOL

PHYLUM	CLASS	ORDER	FAMILY
	Gp1	Gp1	Gp1
	Gp11 BS>Rh	Gp11 BS>Rh	Gp11 BS>Rh
	Gp2	Gp2	Gp2
	Gp22 BS>Rh	Gp22 BS>Rh	Gp22 BS>Rh
	Gp25 BS>Rh	Gp25 BS>Rh	Gp25 BS>Rh
Acidobacteria BS>Rh	Gp3	Gp3	Gp3
	Gp4	Gp4	Gp4
	Gp5 BS>Rh	Gp5 BS>Rh	Gp5 BS>Rh
	Gp6	Gp6	Gp6
	Gp7	Gp7	Gp7
	Holophagae ND	Holophagales ND	Holophagaceae ND
	Unclassified BS>Rh	Unclassified BS>Rh	Unclassified BS>Rh
			Acidimicrobiaceae
		Acidimicrobiales	Iamiaceae
			unclassified
			Acidothermaceae
			Actinomycetaceae
			Actinospicaceae
			Actinosynnemataceae
			Catenulisporaceae
			Cellulomodaceae
			Corynebacteriaceae
			Cryptosporangiaceae
			Geodermatophilaceae
			Glycomycetaceae
			Intrasporangiaceae
			Kineosporiaceae Rh>BS
			Microbacteriaceae
		Actinomycetales	Micrococcaceae
Actinobacteria	Actinobacteria		Micromonosporaceae Rh>BS
			Mycobacteriaceae
			Nakamurellaceae
			Nocardiaceae
			Nocardioidaceae
			Promicromonosporaceae
			Propionibacteriaceae
			Pseudonocardiaceae
			Sporichthyaceae
			Streptomycetaceae Rh>BS
			Streptosporangiaceae
			Thermomonosporaceae
			unclassified
		Bifidobacteriales ND	Bifidobacteriaceae ND
			Conexibacteraceae
		Solirubrobacterales	Solirubrobacteraceae
			unclassified
		unclassified	unclassified
	Flavobacteria	Flavobacteriales	Cryomorphaceae
			Flavobacteriaceae
			Chitinophagaceae BS>Rh
Bacteroidetes BS>Rh	Sphingobacteria BS>Rh	Sphingobacteriales BS>Rh	Cytophagaceae BS>Rh
			Sphingobacteriaceae
			unclassified
	unclassified	unclassified	unclassified
Chlamydiae	Chlamydiae	Chlamydiales	Parachlamydiaceae
			unclassified
Chloroflexi	Chloroflexi	Unclassified	Chloroflexi
	unclassified BS>Rh	unclassified BS>Rh	unclassified BS>Rh
Deinococcus	Deinococci	Deinococcales	Deinococcaceae
			Alicyclobacillaceae ND
			Bacillaceae
			Paenibacillaceae
		Bacillales	Planococcaceae
	Bacilli		Staphylococcaceae
			Thermoactinomycetaceae
			unclassified
		Lactobacillales	Enterococcaceae
Firmicutes			Streptococcaceae ND
		unclassified ND	unclassified ND
			Clostridiaceae
			Gracilibacteraceae ND
			Lachnospiraceae
	Clostridia	Clostridiales	Peptostreptococcaceae
			Ruminococcaceae
			unclassified
			Veillonellaceae ND
	unclassified	unclassified	unclassified
Gemmatimodetes	Gemmatimodetes	Gemmatimodales	Gemmatimodaceae
Nitrospira BS>Rh	Nitrospira BS>Rh	Nitrospirales BS>Rh	Nitrospiraceae BS>Rh

EUTRIC CAMBISOL

PHYLUM	CLASS	ORDER	FAMILY
	Gp1	Gp1	Gp1
	Gp11 ND	Gp11 ND	Gp11 ND
	Gp2 BS>Rh	Gp2 BS>Rh	Gp2 BS>Rh
	Gp22	Gp22	Gp22
	Gp25 ND	Gp25 ND	Gp25 ND
Acidobacteria	Gp3	Gp3	Gp3
	Gp4	Gp4	Gp4
	Gp5	Gp5	Gp5
	Gp6	Gp6	Gp6
	Gp7	Gp7	Gp7
	Holophagae	Holophagales	Holophagaceae
	unclassified	unclassified	unclassified
			Acidimicrobiaceae ND
		Acidimicrobiales	Iamiaceae
			unclassified
			Acidothermaceae
			Actinomycetaceae
			Actinospicaceae Rh>BS
			Actinosynnemataceae
			Catenulisporaceae
			Cellulomonadaceae
			Corynebacteriaceae
			Cryptosporangiaceae
			Geodermatophilaceae ND
			Glycomycetaceae
			Intrasporangiaceae
			Kineosporiaceae Rh>BS
			Microbacteriaceae Rh>BS
		Actinomycetales Rh>BS	Micrococcaceae
Actinobacteria Rh>BS	Actinobacteria Rh>BS		Micromonosporaceae Rh>BS
			Mycobacteriaceae Rh>BS
			Nakamurellaceae
			Nocardiaceae Rh>BS
			Nocardioidaceae
			Promicromonosporaceae ND
			Propionibacteriaceae ND
			Pseudonocardiaceae Rh>BS
			Sporichthyaceae
			Streptomycetaceae Rh>BS
			Streptosporangiaceae
			Thermomonosporaceae
			unclassified Rh>BS
		Bifidobacteriales	Bifidobacteriaceae
			Conexibacteraceae
		Solirubrobacterales	Solirubrobacteraceae
			unclassified
		unclassified	unclassified
	Flavobacteria	Flavobacteriales	Cryomorphaceae ND
			Flavobacteriaceae
			Chitinophagaceae
Bacteroidetes	Sphingobacteria	Sphingobacteriales	Cytophagaceae
			Sphingobacteriaceae Rh>BS
			unclassified
	unclassified	unclassified	unclassified
Chlamydiae	Chlamydiae	Chlamydiales	Parachlamydiaceae
			unclassified
Chloroflexi	Chloroflexi	unclassified	unclassified
	unclassified	unclassified	unclassified
Deinococcus ND	Deinococci ND	Deinococcales ND	Deinococcaceae ND
			Alicyclobacillaceae ND
			Bacillaceae Rh>BS
			Paenibacillaceae
		Bacillales Rh>BS	Planococcaceae
	Bacilli Rh>BS		Staphylococcaceae ND
			Thermoactinomycetaceae ND
			unclassified ND
		Lactobacillales	Enterococcaceae ND
Firmicutes Rh>BS			Streptococcaceae
		unclassified	unclassified
			Clostridiaceae
			Gracilibacteraceae
			Lachnospiraceae ND
	Clostridia	Clostridiales	Peptostreptococcaceae ND
			Ruminococcaceae ND
			unclassified
			Veillonellaceae
	unclassified	unclassified	unclassified
Gemmatimonadetes	Gemmatimonadetes	Gemmatimonadales	Gemmatimonadaceae
Nitrospira	Nitrospira	Nitrospirales	Nitrospiraceae

HYPERDYSTRIC CAMBISOL

PHYLUM	CLASS	ORDER	FAMILY
	Gp1	Gp1	Gp1
	Gp11	Gp11	Gp11
	Gp2	Gp2	Gp2
	Gp22 ND	Gp22 ND	Gp22 ND
	Gp25 ND	Gp25 ND	Gp25 ND
Acidobacteria	Gp3	Gp3	Gp3
	Gp4 ND	Gp4 ND	Gp4 ND
	Gp5 BS>Rh	Gp5 BS>Rh	Gp5 BS>Rh
	Gp6	Gp6	Gp6
	Gp7	Gp7	Gp7
	Holophagae	Holophagales	Holophagaceae
	unclassified	unclassified	unclassified
			Acidimicrobiaceae
		Acidimicrobiales	Iamiaceae
			unclassified
			Acidothermaceae
			Actinomycetaceae
			Actinospicaceae Rh>BS
			Actinosynnemataceae
			Catenulisporaceae Rh>BS
			Cellulomonadaceae
			Corynebacteriaceae
			Cryptosporangiaceae
			Geodermatophilaceae
			Glycomycetaceae
			Intrasporangiaceae
			Kineosporiaceae
			Microbacteriaceae
		Actinomycetales Rh>BS	Micrococcaceae
Actinobacteria Rh>BS	Actinobacteria Rh>BS		Micromonosporaceae Rh>BS
			Mycobacteriaceae Rh>BS
			Nakamurellaceae
			Nocardiaceae Rh>BS
			Nocardioidaceae Rh>BS
			Promicromonosporaceae
			Propionibacteriaceae
			Pseudonocardiaceae
			Sporichthyaceae
			Streptomycetaceae
			Streptosporangiaceae
			Thermomonosporaceae
			unclassified Rh>BS
		Bifidobacteriales ND	Bifidobacteriaceae ND
			Conexibacteraceae
		Solirubrobacterales	Solirubrobacteraceae
			unclassified
		unclassified	unclassified
	Flavobacteria	Flavobacteriales	Cryomorphaceae
			Flavobacteriaceae
			Chitinophagaceae Rh>BS
Bacteroidetes Rh>BS	Sphingobacteria Rh>BS	Sphingobacteriales Rh>BS	Cytophagaceae
			Sphingobacteriaceae Rh>BS
			unclassified
	unclassified	unclassified	unclassified
Chlamydiae	Chlamydiae	Chlamydiales	Parachlamydiaceae
			unclassified
Chloroflexi BS>Rh	Chloroflexi	unclassified	unclassified
	unclassified BS>Rh	unclassified BS>Rh	unclassified BS>Rh
Deinococcus ND	Deinococci ND	Deinococcales ND	Deinococcaceae ND
			Alicyclobacillaceae
			Bacillaceae
			Paenibacillaceae
		Bacillales	Planococcaceae
	Bacilli		Staphylococcaceae
			Thermoactinomycetaceae
			unclassified
		Lactobacillales	Enterococcaceae
Firmicutes			Streptococcaceae
		unclassified ND	unclassified ND
			Clostridiaceae
			Gracilibacteraceae
			Lachnospiraceae
	Clostridia	Clostridiales	Peptostreptococcaceae
			Ruminococcaceae
			unclassified
			Veillonellaceae
	unclassified	unclassified	unclassified
Gemmatimonadetes	Gemmatimonadetes	Gemmatimonadales	Gemmatimonadaceae
Nitrospira BS>Rh	Nitrospira BS>Rh	Nitrospirales BS>Rh	Nitrospiraceae BS>Rh

Table.S4 Part 2: Changes in taxonomic composition between bulk soil and rhizosphere samples for each soil type. Text in red and blue indicates a significant increase in rhizosphere and bulks soils samples respectively. Samples are referred as follow: BS: Bulk Soil; Rh: Rhizosphere. The symbols '>' and '<' mean significantly more or less abundant and '=' not significantly different.

CALCARIC CAMBISOL

PHYLUM	CLASS	ORDER	FAMILY
OP10	OP10	OP10	OP10
Planctomycetes	Planctomycetes	Planctomycetales	Planctomycetaceae
			Caulobacteraceae Rh>BS
		Caulobacterales Rh>BS	Hyphomodaceae Rh>BS
			unclassified
			Beijerinckiaceae
			Bradyrhizobiaceae
			Brucellaceae
			Hyphomicrobiaceae
			Methylobacteriaceae
		Rhizobiales	Methylocystaceae
			Phyllobacteriaceae Rh>BS
	Alpha-proteobacteria		Rhizobiaceae
			Rhodobiaceae
			unclassified
			Xanthobacteraceae
		Rhodobacterales Rh>BS	Rhodobacteraceae Rh>BS
			Acetobacteraceae
		Rhodospirillales	Rhodospirillaceae
			unclassified
		Rickettsiales	Rickettsiaceae
		Sphingomodales	Sphingomodaceae
			unclassified
		unclassified	unclassified
			Alcaligeceae
			Burkholderiaceae
		Burkholderiales Rh>BS	Burkholderiales_incertae_sedis Rh>BS
			Comamodaceae Rh>BS
			Oxalobacteraceae
Proteobacteria	Beta-proteobacteria		unclassified
		Methylophilales	Methylophilaceae
		Neisseriales	Neisseriaceae
		Nitrosomodales	Nitrosomodaceae
		Rhodocyclales Rh>BS	Rhodocyclaceae Rh>BS
		unclassified BS>Rh	unclassified BS>Rh
		Bdellovibrionales BS>Rh	Bacteriovoracaceae
			Bdellovibrioceae
		Desulfobacterales	unclassified
		Desulfuromodales	Geobacteraceae
			unclassified
	Delta-proteobacteria BS>Rh		Cystobacteraceae
			Haliangiaceae
		Myxococcales BS>Rh	Kofleriaceae
			nnocystaceae
			Polyangiaceae
			unclassified BS>Rh
		unclassified BS>Rh	unclassified BS>Rh
		Aeromodales	Aeromodaceae
		Alteromodales	Alteromodaceae
		Chromatiales	unclassified
		Enterobacteriales	Enterobacteriaceae
	Gamma-proteobacteria	Pseudomodales	Moraxellaceae
			Pseudomodaceae
		unclassified	unclassified
			Sinobacteraceae
		Xanthomodales	unclassified
			Xanthomodaceae
	unclassified	unclassified	unclassified
			Leptospiraceae
Spirochaetes Rh>BS	Spirochaetes Rh>BS	Spirochaetales Rh>BS	Spirochaetaceae
			unclassified
TM7 Rh>BS	TM7 Rh>BS	TM7 Rh>BS	TM7 Rh>BS
	Opitutae	Opitutales	Opitutaceae
Verrucomicrobia	Spartobacteria	Spartobacteria	Spartobacteria
	Subdivision3	Subdivision3	Subdivision3
	unclassified	unclassified	unclassified
WS3	WS3	WS3	unclassified

EUTRIC CAMBISOL

PHYLUM	CLASS	ORDER	FAMILY
OP10	OP10	genera	incertae
Planctomycetes ND	Planctomycetacia ND	Planctomycetales ND	Planctomycetaceae ND
			Caulobacteraceae
		Caulobacterales	Hyphomonadaceae
			unclassified
			Beijerinckiaceae Rh>BS
			Bradyrhizobiaceae Rh>BS
			Brucellaceae ND
			Hyphomicrobiaceae
			Methylobacteriaceae ND
		Rhizobiales Rh>BS	Methylocystaceae
			Phyllobacteriaceae Rh>BS
	Alpha-proteobacteria		Rhizobiaceae Rh>BS
			Rhodobiaceae
			unclassified
			Xanthobacteraceae
		Rhodobacterales	Rhodobacteraceae
			Acetobacteraceae
		Rhodospirillales	Rhodospirillaceae Rh>BS
			unclassified
		Rickettsiales	Rickettsiaceae
		Sphingomonadales	Sphingomonadaceae
			unclassified
		unclassified	unclassified
			Alcaligenaceae ND
			Burkholderiaceae Rh>BS
		Burkholderiales Rh>BS	Burkholderiales_incertae_sedis Rh>BS
			Comamonadaceae
			Oxalobacteraceae Rh>BS
Proteobacteria	Beta-proteobacteria Rh>BS		unclassified Rh>BS
		Methylophilales	Methylophilaceae
		Neisseriales ND	Neisseriaceae ND
		Nitrosomonadales	Nitrosomonadaceae
		Rhodocyclales	Rhodocyclaceae
		unclassified	unclassified
		Bdellovibrionales	Bacteriovoracaceae
			Bdellovibrionaceae
		Desulfobacterales ND	unclassified ND
		Desulfuromonadales	Geobacteraceae
			unclassified ND
	Delta-proteobacteria		Cystobacteraceae
			Haliangiaceae ND
		Myxococcales	Kofleriaceae
			Nannocystaceae ND
			Polyangiaceae
			unclassified
		unclassified	unclassified
		Aeromonadales	Aeromonadaceae
		Alteromonadales ND	Alteromonadaceae ND
		Chromatiales ND	unclassified ND
		Enterobacteriales	Enterobacteriaceae
	Gamma-proteobacteria	Pseudomonadales	Moraxellaceae
			Pseudomonadaceae
		unclassified	unclassified
			Sinobacteraceae
		Xanthomonadales	unclassified
			Xanthomonadaceae
	unclassified	unclassified	unclassified
			Leptospiraceae ND
Spirochaetes	Spirochaetes	Spirochaetales	Spirochaetaceae ND
			unclassified
TM7 Rh>BS	TM7 Rh>BS	TM7 Rh>BS	TM7 Rh>BS
	Opitutae ND	Opitutales ND	Opitutaceae ND
Verrucomicrobia	Spartobacteria	Spartobacteria	Spartobacteria
	Subdivision3	Subdivision3	Subdivision3
	unclassified ND	unclassified ND	unclassified ND
WS3 ND	WS3 ND	WS3 ND	WS3 ND

HYPERDYSTRIC CAMBISOL

PHYLUM	CLASS	ORDER	FAMILY
OP10	OP10	genera	incertae
Planctomycetes ND	Planctomycetacia ND	Planctomycetales ND	Planctomycetaceae ND
			Caulobacteraceae Rh>BS
		Caulobacterales Rh>BS	Hyphomonadaceae
			unclassified
			Beijerinckiaceae Rh>BS
			Bradyrhizobiaceae Rh>BS
			Brucellaceae
			Hyphomicrobiaceae
			Methylobacteriaceae
		Rhizobiales	Methylocystaceae
			Phyllobacteriaceae
	Alpha-proteobacteria		Rhizobiaceae Rh>BS
			Rhodobiaceae
			unclassified
			Xanthobacteraceae Rh>BS
		Rhodobacterales	Rhodobacteraceae
			Acetobacteraceae
		Rhodospirillales	Rhodospirillaceae Rh>BS
			unclassified
		Rickettsiales	Rickettsiaceae
		Sphingomonadales	Sphingomonadaceae
			unclassified
		unclassified	unclassified
			Alcaligenaceae
			Burkholderiaceae Rh>BS
		Burkholderiales Rh>BS	Burkholderiales_incertae_sedis Rh>BS
			Comamonadaceae
			Oxalobacteraceae
Proteobacteria	Beta-proteobacteria		unclassified
		Methylophilales	Methylophilaceae
		Neisseriales	Neisseriaceae
		Nitrosomonadales	Nitrosomonadaceae
		Rhodocyclales	Rhodocyclaceae
		unclassified	unclassified
		Bdellovibrionales	Bacteriovoracaceae
			Bdellovibrionaceae
		Desulfobacterales ND	unclassified ND
		Desulfuromonadales ND	Geobacteraceae ND
			unclassified ND
	Delta-proteobacteria		Cystobacteraceae
			Haliangiaceae
		Myxococcales	Kofleriaceae
			Nannocystaceae
			Polyangiaceae
			unclassified
		unclassified	unclassified
		Aeromonadales ND	Aeromonadaceae ND
		Alteromonadales ND	Alteromonadaceae ND
		Chromatiales ND	unclassified ND
		Enterobacteriales	Enterobacteriaceae
	Gamma-proteobacteria	Pseudomonadales	Moraxellaceae
			Pseudomonadaceae
		unclassified	unclassified
			Sinobacteraceae
		Xanthomonadales	unclassified
			Xanthomonadaceae Rh>BS
	unclassified Rh>BS	unclassified Rh>BS	unclassified Rh>BS
			Leptospiraceae ND
Spirochaetes ND	Spirochaetes ND	Spirochaetales ND	Spirochaetaceae ND
			unclassified ND
TM7	TM7	TM7	TM7
	Opitutae ND	Opitutales ND	Opitutaceae ND
Verrucomicrobia	Spartobacteria	Spartobacteria	Spartobacteria
	Subdivision3	Subdivision3	Subdivision3
	unclassified	unclassified	unclassified
WS3 ND	WS3 ND	WS3 ND	WS3 ND

Table.S5 Relative abundance (in %) of the bacterial genera that constituted the rhizosphere/core OTUs along the soil toposequence. Each value is the mean of biological replicates (± Standard Error of the Mean).

Phylum	Class	Order	Family	Genus	Calcaric		Eutric		Hyperdystric	
					mean	sem	mean	sem	mean	sem
Acidobacteria	Acidobacteria_Gp2	Gp2	unclassified	unclassified	0.61	0.35	17.30	2.76	17.93	1.62
Acidobacteria	Acidobacteria_Gp3	Gp3	unclassified	unclassified	1.89	0.34	4.44	0.38	6.45	0.79
Actinobacteria	Actinobacteria	Acidimicrobiales	Acidimicrobineae	unclassified	3.97	0.47	0.37	0.13	0.58	0.07
Actinobacteria	Actinobacteria	Acidimicrobiales	Iamiaceae	Iamia	0.24	0.11	0.09	0.01	0.52	0.28
Actinobacteria	Actinobacteria	Acidimicrobiales	unclassified	unclassified	1.00	0.13	0.45	0.17	0.14	0.05
Actinobacteria	Actinobacteria	Actinomycetales	Micromonosporaceae	unclassified	0.87	0.14	0.17	0.04	0.45	0.14
Actinobacteria	Actinobacteria	Actinomycetales	Mycobacteriaceae	Mycobacterium	23.40	6.42	15.14	2.35	7.99	1.01
Actinobacteria	Actinobacteria	Actinomycetales	Streptomycetaceae	unclassified	2.43	0.59	0.79	0.24	0.91	0.51
Actinobacteria	Actinobacteria	Actinomycetales	Streptosporangineae	unclassified	0.12	0.05	0.16	0.04	0.17	0.04
Actinobacteria	Actinobacteria	Actinomycetales	unclassified	unclassified	0.91	0.20	17.81	2.20	20.92	2.72
Actinobacteria	Actinobacteria	Solirubrobacterales	Conexibacteraceae	unclassified	0.75	0.16	1.87	0.60	0.70	0.12
Actinobacteria	Actinobacteria	Solirubrobacterales	unclassified	unclassified	4.24	0.98	2.39	1.16	0.24	0.07
Actinobacteria	Actinobacteria	unclassified	unclassified	unclassified	4.70	0.84	3.52	0.77	2.00	0.46
Bacteroidetes	Sphingobacteria	Sphingobacteriales	Chitinophagaceae	unclassified	1.28	0.41	1.38	0.33	2.85	0.79
Bacteroidetes	Sphingobacteria	Sphingobacteriales	Cytophagaceae	unclassified	0.56	0.06	0.46	0.17	0.47	0.14
Chloroflexi	unclassified_Chloroflexi	unclassified	unclassified	unclassified	0.52	0.11	0.08	0.03	0.18	0.07
Nitrospira	Nitrospira	Nitrospirales	Nitrospiraceae	Nitrospira	0.33	0.05	0.34	0.06	1.50	0.08
Proteobacteria	Alphaproteobacteria	Caulobacterales	Caulobacteraceae	Phenylobacterium	1.93	0.38	1.69	0.15	1.44	0.19
Proteobacteria	Alphaproteobacteria	Caulobacterales	Caulobacteraceae	unclassified	0.21	0.06	0.58	0.17	1.37	0.08
Proteobacteria	Alphaproteobacteria	Rhizobiales	Bradyrhizobiaceae	Bradyrhizobium	10.45	1.88	9.75	1.44	9.23	1.17
Proteobacteria	Alphaproteobacteria	Rhizobiales	Bradyrhizobiaceae	unclassified	0.39	0.33	0.26	0.08	0.26	0.05
Proteobacteria	Alphaproteobacteria	Rhizobiales	Hyphomicrobiaceae	Rhodoplanes	12.26	1.79	1.04	0.11	0.52	0.11
Proteobacteria	Alphaproteobacteria	Rhizobiales	Hyphomicrobiaceae	unclassified	0.78	0.16	0.30	0.04	0.71	0.08
Proteobacteria	Alphaproteobacteria	Rhizobiales	unclassified_Rhizobiales	unclassified	0.87	0.28	1.07	0.28	1.62	0.09
Proteobacteria	Alphaproteobacteria	Rhizobiales	unclassified	unclassified	0.81	0.32	2.02	0.41	2.19	0.52
Proteobacteria	Alphaproteobacteria	Rhizobiales	Xanthobacteraceae	Pseudolabrys	0.56	0.27	0.32	0.19	0.30	0.17
Proteobacteria	Alphaproteobacteria	Rhodospirillales	unclassified	unclassified	1.41	0.58	2.07	0.25	1.45	0.26
Proteobacteria	Alphaproteobacteria	unclassified	unclassified	unclassified	7.24	1.03	1.71	0.22	1.74	0.35
Proteobacteria	Betaproteobacteria	unclassified	unclassified	unclassified	0.33	0.11	0.33	0.03	0.09	0.02
Proteobacteria	Deltaproteobacteria	Myxococcales	Cystobacterineae	unclassified	0.53	0.13	0.13	0.03	0.55	0.07
Proteobacteria	Deltaproteobacteria	Myxococcales	Sorangiineae	Polyangiaceae	1.83	0.44	0.23	0.03	0.49	0.06
Proteobacteria	Deltaproteobacteria	Myxococcales	Sorangiineae	unclassified	0.76	0.13	0.25	0.02	0.36	0.06
Proteobacteria	Deltaproteobacteria	unclassified	unclassified	unclassified	3.46	0.51	0.70	0.13	0.41	0.07
Proteobacteria	Gammaproteobacteria	Xanthomonadales	Sinobacteraceae	Steroidobacter	0.86	0.28	5.61	0.82	7.87	0.84
Proteobacteria	Gammaproteobacteria	Xanthomonadales	Xanthomonadaceae	unclassified	0.82	0.39	0.87	0.22	0.10	0.03
Proteobacteria	unclassified	unclassified	unclassified	unclassified	2.43	0.54	1.96	0.48	2.53	0.63
unclassified	unclassified	unclassified	unclassified	unclassified	2.32	0.38	0.87	0.08	1.93	0.38
Verrucomicrobia	Subdivision3	Subdivision3	unclassified	unclassified	1.93	0.32	1.51	0.22	0.83	0.13

Table.S6 Relative abundance (in %) of the bacterial genera that constituted the acidic rhizosphere/core OTUs in Cambisol Eutric and Hyperdystric.

Each value is the mean of biological replicates (± Standard Error of the Mean).

Phylum	Class	Order	Family	Genus	Eutric		Hyperdystric	
					mean	sem	mean	sem
Acidobacteria	Acidobacteria_Gp1	Gp1	unclassified	unclassified	7.1619708	1.26508868	4.27473571	0.76908305
Acidobacteria	Acidobacteria_Gp2	Gp2	unclassified	unclassified	10.7687284	1.69642531	9.65769631	2.20669787
Acidobacteria	Acidobacteria_Gp3	Gp3	unclassified	unclassified	3.92180598	0.14366937	3.75323336	0.87623791
Acidobacteria	unclassified	unclassified	unclassified	unclassified	2.29702904	0.52333814	2.01737297	0.54886993
Actinobacteria	Actinobacteria	Acidimicrobiales	Iamiaceae	Iamia	0.94281404	0.11548958	0.82031669	0.13818892
Actinobacteria	Actinobacteria	Acidimicrobiales	unclassified	unclassified	1.32951199	0.15983938	1.10607781	0.0470451
Actinobacteria	Actinobacteria	Actinomycetales	Actinospicaceae	Actinospica	2.19872497	0.32889383	6.45704419	2.2678257
Actinobacteria	Actinobacteria	Actinomycetales	Catenulisporaceae	Catenulispora	0.74501019	0.26889465	0.77948347	0.16668271
Actinobacteria	Actinobacteria	Actinomycetales	Micromonosporaceae	unclassified	0.07408839	0.02259253	0.11129536	0.03648324
Actinobacteria	Actinobacteria	Actinomycetales	Mycobacteriaceae	Mycobacterium	6.4638048	1.19515787	2.06165288	0.20265768
Actinobacteria	Actinobacteria	Actinomycetales	Streptomycetaceae	Streptacidiphilus	2.9649012	0.40010128	7.0291416	3.50255433
Actinobacteria	Actinobacteria	Actinomycetales	Streptomycetaceae	unclassified	0.32932238	0.09226806	0.21670263	0.11662585
Actinobacteria	Actinobacteria	Actinomycetales	unclassified	unclassified	10.6838015	1.11448578	7.77892647	0.4149742
Actinobacteria	Actinobacteria	Solirubrobacterales	Conexibacteraceae	Conexibacter	2.176975	0.51994115	0.59076219	0.10718727
Actinobacteria	Actinobacteria	Solirubrobacterales	unclassified	unclassified	1.12834373	0.46229552	0.13496295	0.01234333
Actinobacteria	Actinobacteria	unclassified	unclassified	unclassified	1.86598982	0.37060741	0.76111444	0.11055591
Bacteroidetes	Sphingobacteria	Sphingobacteriales	Chitinophagaceae	unclassified	1.53953432	0.24013384	2.27687827	0.39785909
Bacteroidetes	Sphingobacteria	Sphingobacteriales	Cytophagaceae	unclassified	0.20321017	0.08142207	0.12867373	0.0438941
Bacteroidetes	Sphingobacteria	Sphingobacteriales	Sphingobacteriaceae	unclassified	0.42013405	0.08136681	0.60118857	0.06694591
Chloroflexi	unclassified_Chloroflexi	unclassified	unclassified	unclassified	0.03359409	0.01179497	0.04358075	0.01367039
Gemmatimonadetes	Gemmatimonadetes	Gemmatimonadales	Gemmatimonadaceae	Gemmatimonas	0.21175362	0.03619133	0.1133986	0.03639352
Nitrospira	Nitrospira	Nitrospirales	Nitrospiraceae	Nitrospira	0.14642928	0.02771709	0.40262733	0.05605768
OP10	OP10_genera_incertae_sedis	unclassified	unclassified	unclassified	0.03950047	0.00799948	0.05950277	0.01894935
Proteobacteria	Alphaproteobacteria	Caulobacterales	Caulobacteraceae	Phenylobacterium	0.71866159	0.08445406	0.38543977	0.07804105
Proteobacteria	Alphaproteobacteria	Caulobacterales	Caulobacteraceae	unclassified	0.27666577	0.07785411	0.38305028	0.05257617
Proteobacteria	Alphaproteobacteria	Rhizobiales	Beijerinckiaceae	unclassified	0.17879214	0.02629573	0.35904672	0.1073096
Proteobacteria	Alphaproteobacteria	Rhizobiales	Bradyrhizobiaceae	Bradyrhizobium	4.05890381	0.51088834	2.52356078	0.55834465
Proteobacteria	Alphaproteobacteria	Rhizobiales	Bradyrhizobiaceae	unclassified	0.22283857	0.04427219	0.15402185	0.03720198
Proteobacteria	Alphaproteobacteria	Rhizobiales	Hyphomicrobiaceae	Rhodoplanes	0.44172633	0.05529381	0.14292912	0.04561979
Proteobacteria	Alphaproteobacteria	Rhizobiales	Hyphomicrobiaceae	unclassified	0.12948631	0.0221265	0.19393693	0.03695086
Proteobacteria	Alphaproteobacteria	Rhizobiales	Rhizobiaceae	Rhizobium	0.23367647	0.04889463	0.22006675	0.05441965
Proteobacteria	Alphaproteobacteria	Rhizobiales	unclassified	unclassified	2.86952085	0.23628056	2.98304448	0.96975125
Proteobacteria	Alphaproteobacteria	Rhizobiales	Xanthobacteraceae	Labrys	0.25480139	0.06688175	0.07262743	0.0155729
Proteobacteria	Alphaproteobacteria	Rhizobiales	Xanthobacteraceae	Pseudolabrys	0.15162902	0.09692308	0.0841106	0.0492838
Proteobacteria	Alphaproteobacteria	Rhodospirillales	Acetobacteraceae	unclassified	0.58017519	0.09088377	0.46613032	0.13510524
Proteobacteria	Alphaproteobacteria	Rhodospirillales	unclassified	unclassified	2.48951269	0.29920027	1.95611745	0.48003991
Proteobacteria	Alphaproteobacteria	unclassified	unclassified	unclassified	2.2069181	0.15698593	1.91379023	0.35869423
Proteobacteria	Betaproteobacteria	Burkholderiales	Burkholderiaceae	Burkholderia	0.992053	0.18199842	1.24394895	0.28202441
Proteobacteria	Betaproteobacteria	Burkholderiales	Burkholderiales_incertae_sedis	unclassified	0.20401604	0.05992309	0.59756128	0.18325457
Proteobacteria	Betaproteobacteria	Burkholderiales	Oxalobacteraceae	Collimonas	0.15319064	0.04891661	0.31756524	0.14640665
Proteobacteria	Betaproteobacteria	Burkholderiales	Oxalobacteraceae	Herbaspirillum	0.01939039	0.00505322	0.058756	0.02729537
Proteobacteria	Betaproteobacteria	unclassified	unclassified	unclassified	0.25552117	0.0441768	0.07587177	0.01621753
Proteobacteria	Deltaproteobacteria	Myxococcales	Polyangiaceae	unclassified	0.57167185	0.1151067	0.76041825	0.1959481
Proteobacteria	Deltaproteobacteria	Myxococcales	unclassified	unclassified	0.20876274	0.0306421	0.32626043	0.05238568
Proteobacteria	Deltaproteobacteria	unclassified	unclassified	unclassified	0.87364326	0.14526787	0.58954662	0.14488548
Proteobacteria	Gammaproteobacteria	Xanthomonadales	Sinobacteraceae	Nevskia	0.04926228	0.02226176	0.19745547	0.03426669
Proteobacteria	Gammaproteobacteria	Xanthomonadales	Sinobacteraceae	Steroidobacter	5.44268369	0.80333849	5.38357838	1.34269212
Proteobacteria	Gammaproteobacteria	Xanthomonadales	Xanthomonadaceae	unclassified	0.81742254	0.21150983	0.3287248	0.06761882
Proteobacteria	unclassified	unclassified	unclassified	unclassified	0.80212447	0.18758711	0.71780224	0.24127958
TM7	TM7_genera_incertae_sedis	unclassified	unclassified	unclassified	0.33605932	0.13612436	2.62743007	1.09875336
unclassified	unclassified	unclassified	unclassified	unclassified	15.6211728	3.11325192	23.0291592	6.49041075
Verrucomicrobia	Subdivision3	unclassified	unclassified	unclassified	1.19273931	0.17086568	0.73167955	0.17550021

Table S7: Relative distribution of the functional categories of the Geochip in phyla and major classes according to the compartment (Rhizosphere, Rh and Bulk soil, BS) and the soil type (Calcaric, Eutric and Hyperdistric).

	Relative signal expressed in %						Distribution in classes (a,b,c) according to the p values					
	**Calcaric**		**Eutric**		**Hyperdistric**		**Calcaric**		**Eutric**		**Hyperdistric**	
**Carbon cycling**	**BS**	**Rh**	**BS**	**Rh**	**BS**	**Rh**	**BS**	**Rh**	**BS**	**Rh**	**BS**	**Rh**
Actinobacteria	4.43	4.20	4.16	4.23	4.41	4.26	b	a	a	a	b	a
Alphaproteobacteria	2.30	2.27	2.31	2.26	2.30	2.27	a	a	a	a	a	a
Gammaproteobacteria	1.27	1.35	1.33	1.32	1.26	1.33	b	a	a	a	b	a
Firmicutes	0.86	0.89	0.92	0.90	0.87	0.90	b	ab	a	ab	b	ab
Betaproteobacteria	0.87	0.90	0.89	0.89	0.88	0.89	b	a	ab	ab	ab	ab
	**Calcaric**		**Eutric**		**Hyperdistric**		**Calcaric**		**Eutric**		**Hyperdistric**	
**Metal _homeostasis**	**BS**	**Rh**	**BS**	**Rh**	**BS**	**Rh**	**BS**	**Rh**	**BS**	**Rh**	**BS**	**Rh**
Alphaproteobacteria	6.58	6.56	6.50	6.50	6.58	6.58	b	ab	a	a	b	b
Actinobacteria	5.26	5.13	5.06	5.09	5.30	5.07	b	a	a	a	b	a
Gammaproteobacteria	4.09	4.26	4.36	4.36	4.16	4.26	d	ab	c	ac	bd	abc
Betaproteobacteria	3.72	3.70	3.66	3.63	3.69	3.65	b	ab	ab	a	ab	ab
Firmicutes	1.40	1.53	1.56	1.53	1.38	1.55	b	a	a	a	b	a
	**Calcaric**		**Eutric**		**Hyperdistric**		**Calcaric**		**Eutric**		**Hyperdistric**	
**Nitrogen**	**BS**	**Rh**	**BS**	**Rh**	**BS**	**Rh**	**BS**	**Rh**	**BS**	**Rh**	**BS**	**Rh**
Actinobacteria	0.48	0.46	0.43	0.43	0.47	0.45	a	a	a	a	a	a
Alphaproteobacteria	0.26	0.26	0.26	0.27	0.27	0.26	a	a	a	a	a	a
Betaproteobacteria	0.26	0.25	0.26	0.26	0.26	0.26	a	a	a	a	a	a
Gammaproteobacteria	0.23	0.23	0.24	0.24	0.23	0.24	a	a	ab	b	a	ab
Firmicutes	0.10	0.12	0.12	0.12	0.10	0.12	b	a	a	a	b	a
	**Calcaric**		**Eutric**		**Hyperdistric**		**Calcaric**		**Eutric**		**Hyperdistric**	
**Organic_remediation**	**BS**	**Rh**	**BS**	**Rh**	**BS**	**Rh**	**BS**	**Rh**	**BS**	**Rh**	**BS**	**Rh**
Alphaproteobacteria	2.51	2.48	2.48	2.51	2.48	2.53	ab	a	a	ab	a	b
Betaproteobacteria	1.69	1.70	1.64	1.67	1.67	1.69	a	a	b	ab	ab	a
Actinobacteria	1.41	1.32	1.32	1.33	1.42	1.33	b	a	a	a	b	a
Gammaproteobacteria	1.01	1.06	1.07	1.05	1.03	1.05	c	ab	a	ab	bc	abc
Firmicutes	0.18	0.19	0.20	0.20	0.18	0.20	c	abc	b	ab	ac	ab
	**Calcaric**		**Eutric**		**Hyperdistric**		**Calcaric**		**Eutric**		**Hyperdistric**	
**Phosphorus**	**BS**	**Rh**	**BS**	**Rh**	**BS**	**Rh**	**BS**	**Rh**	**BS**	**Rh**	**BS**	**Rh**
Alphaproteobacteria	0.48	0.47	0.48	0.47	0.49	0.47	a	a	a	a	a	a
Actinobacteria	0.47	0.45	0.44	0.46	0.48	0.46	a	a	a	a	a	a
Gammaproteobacteria	0.26	0.25	0.26	0.27	0.26	0.26	a	a	ab	b	ab	ab
Betaproteobacteria	0.20	0.20	0.20	0.20	0.21	0.20	a	a	a	a	a	a
Firmicutes	0.10	0.11	0.11	0.11	0.10	0.11	a	a	a	a	a	a

	Calcaric		Eutric		Hyperdistric		Calcaric		Eutric		Hyperdistric	
**Secondary_metabolism**	**BS**	**Rh**	**BS**	**Rh**	**BS**	**Rh**	**BS**	**Rh**	**BS**	**Rh**	**BS**	**Rh**
Actinobacteria	1.19	1.10	1.07	1.10	1.17	1.12	c	ab	a	ab	c	b
Alphaproteobacteria	0.75	0.74	0.74	0.74	0.77	0.73	ab	ab	ab	ab	a	b
Gammaproteobacteria	0.27	0.28	0.29	0.28	0.27	0.28	a	a	a	a	a	a
Betaproteobacteria	0.29	0.27	0.27	0.28	0.29	0.27	b	a	ab	ab	ab	a
Firmicutes	0.06	0.06	0.07	0.07	0.06	0.06	a	a	a	a	a	a
	**Calcaric**		**Eutric**		**Hyperdistric**		**Calcaric**		**Eutric**		**Hyperdistric**	
**Stress**	**BS**	**Rh**	**BS**	**Rh**	**BS**	**Rh**	**BS**	**Rh**	**BS**	**Rh**	**BS**	**Rh**
Alphaproteobacteria	4.50	4.48	4.39	4.40	4.38	4.47	a	a	a	a	a	a
Actinobacteria	3.52	3.42	3.40	3.41	3.51	3.42	b	a	a	a	b	a
Gammaproteobacteria	2.48	2.56	2.57	2.57	2.48	2.57	b	a	a	a	b	a
Betaproteobacteria	1.83	1.83	1.80	1.82	1.86	1.82	a	a	a	a	a	a
Firmicutes	0.91	0.94	0.98	0.96	0.90	0.96	a	a	a	a	a	a
	**Calcaric**		**Eutric**		**Hyperdistric**		**Calcaric**		**Eutric**		**Hyperdistric**	
**Sulfur**	**BS**	**Rh**	**BS**	**Rh**	**BS**	**Rh**	**BS**	**Rh**	**BS**	**Rh**	**BS**	**Rh**
Alphaproteobacteria	0.59	0.57	0.59	0.58	0.60	0.57	cd	ab	cd	ac	d	b
Gammaproteobacteria	0.38	0.40	0.40	0.39	0.39	0.39	b	a	a	ab	ab	ab
Betaproteobacteria	0.27	0.30	0.28	0.29	0.28	0.29	a	a	a	a	a	a
Actinobacteria	0.10	0.10	0.10	0.10	0.11	0.10	a	a	a	a	a	a
Firmicutes	0.07	0.07	0.07	0.07	0.07	0.07	b	ab	ab	ab	ab	a
	**Calcaric**		**Eutric**		**Hyperdistric**		**Calcaric**		**Eutric**		**Hyperdistric**	
**Virulence**	**BS**	**Rh**	**BS**	**Rh**	**BS**	**Rh**	**BS**	**Rh**	**BS**	**Rh**	**BS**	**Rh**
Actinobacteria	8.52	7.95	7.89	7.88	8.46	7.92	b	a	a	a	b	a
Gammaproteobacteria	3.71	3.77	3.79	3.84	3.76	3.74	b	ab	ab	a	ab	b
Alphaproteobacteria	3.19	3.31	3.23	3.23	3.21	3.21	a	a	a	a	a	a
Betaproteobacteria	2.50	2.46	2.46	2.47	2.54	2.44	bc	ab	ab	ab	c	a
Firmicutes	0.33	0.38	0.40	0.38	0.34	0.39	b	a	a	a	b	a
	**Calcaric**		**Eutric**		**Hyperdistric**		**Calcaric**		**Eutric**		**Hyperdistric**	
**CRISPR**	**BS**	**Rh**	**BS**	**Rh**	**BS**	**Rh**	**BS**	**Rh**	**BS**	**Rh**	**BS**	**Rh**
Firmicutes	0.49	0.56	0.55	0.53	0.48	0.54	b	a	a	a	b	a
Actinobacteria	0.45	0.44	0.45	0.45	0.46	0.45	a	a	a	a	a	a
Gammaproteobacteria	0.36	0.37	0.38	0.38	0.36	0.38	c	ab	a	a	bc	a
Alphaproteobacteria	0.29	0.29	0.29	0.29	0.28	0.29	a	a	a	a	a	a
deltaproteobacteria	0.24	0.25	0.26	0.25	0.24	0.25	ab	ab	a	ab	b	ab

Table.S8 Pearson correlation between soil parameters and the relative abundance of bacterial phyla in bulk soil samples. The r-value is provided for significant correlations (**P<0.01 and ***P<0.001).

	pH	Hum	N.total	C.total	C.N	OM	Limestone	ATE	CEC	H.	Ca	K	Mg	Na	P.Duch.	P.Olsen	Mn	Al	Fe
**Acidobacteria**	-0.784**	-0.75**	-0.805**	-0.8**		-0.8**	-0.816**	0.914***	-0.815**	0.841***	-0.83***	-0.765**						0.911***	
**Bacteroidetes**	0.978***	0.974***	0.967***	0.958***		0.958***	0.929***	-0.932***	0.976***	-0.954***	0.974***	0.964***	0.904***	0.937***			-0.749**	-0.914***	
**Chloroflexi**	0.875***	0.884***	0.87***	0.867***		0.867***	0.808**	-0.865***	0.901***	-0.887***	0.908***	0.912***	0.865***	0.785**			-0.708**	-0.846***	
**Firmicutes**	0.939***	0.943***	0.951***	0.942***		0.942***	0.982***	-0.936***	0.958***	-0.951***	0.962***	0.943***	0.872***	0.854***			-0.713**	-0.918***	
**Gemmatimonadetes**	0.769**	0.746**							0.718**	-0.739**	0.712**	0.746**		0.816**					
**Nitrospira**	0.895***	0.863***	0.845***	0.823**		0.823**	0.887***	-0.93***	0.892***	-0.909***	0.901***	0.884***	0.727**	0.773**				-0.922***	
**Alphaproteobacteria**	0.742**	0.75**										0.753**		0.822**					
**Betaproteobacteria**	0.981***	0.963***	0.944***	0.934***		0.934***	0.947***	-0.957***	0.975***	-0.973***	0.979***	0.966***	0.866***	0.912***			-0.727**	-0.939***	
**Deltaproteobacteria**	0.954***	0.938***	0.894***	0.876***		0.875***	0.916***	-0.869***	0.938***	-0.925***	0.938***	0.955***	0.827***	0.91***			-0.789**	-0.845***	
**Gammaproteobacteria**	0.744**	0.754**							0.718**			0.752**		0.803**					
**Proteobacteria uncl.**	0.988***	0.969***	0.947***	0.932***		0.932***	0.96***	-0.945***	0.981***	-0.975***	0.983***	0.979***	0.865***	0.925***			-0.792**	-0.923***	
**Acidimicrobiales**	0.892***	0.88***	0.888***	0.889***		0.889***	0.857***	-0.817**	0.891***	-0.896***	0.886***	0.86***	0.813**	0.842***			-0.809**	-0.777**	
**Actinomycetales**	0.72**		0.732**	0.734**		0.734**								0.715**			-0.714**		
**Solirubrobacterales**	0.947***	0.942***	0.947***	0.946***		0.946***	0.902***	-0.861***	0.941***	-0.935***	0.934***	0.913***	0.874***	0.924***			-0.824***	-0.827***	
**Actinomycetales uncl.**	0.856***	0.863***	0.861***	0.861***		0.862***	0.798**	-0.735**	0.849***	-0.827***	0.837***	0.824***	0.829***	0.847***			-0.79**		
**TM7**													0.718**						
**Verrucomicrobia**														0.71**					
**Unclassified**	-0.847***	-0.855***	-0.795**	-0.787**		-0.787**	-0.746**		-0.814**	0.776**	-0.8**	-0.839***	-0.796**	-0.888***			0.779**		

Table.S9 Pearson correlation between soil parameters and the substrate utilization patterns (Biolog Ecoplates). The r-value is provided for significant correlations (**P<0.01 and ***P<0.001).

	pH	Hum	N.total	C.total	C.N	OM	Limestone	ATE	CEC	H.	Ca	K	Mg	Na	P.Duch.	P.Olsen	Mn	Al	Fe
**pyruvic.acid.methyl.ester**		-0.706**												-0.753**			0.748**		
**tween.40**																			
**tween.80**																			
**alpha.cyclodextrin**	0.879***	0.889***	0.895***	0.886***		0.886***	0.935***	-0.812***	0.883***	-0.848***	0.881***	0.877***	0.834***	0.85***				-0.793**	
**glycogen**	0.861***	0.792**	0.738**	0.721**		0.721**	0.81***	-0.824***	0.82***	-0.876***	0.825***	0.803**		0.77**			-0.749**	-0.791**	
**D.cellubiose**																			
**alpha.D.Lactose**																			
**beta.methyl.D.glucoside**																			
**D.xylose**																			
**i.Erythritol**																			
**D.Mannitol**																			
**N.Acetyl.D.Glucosamine**																			
**D.Glucosaminidic.acid**																			
**Glucose.1.phosphate**																			
**Glycerol.phosphate**																			
**D.galactonique.acid.gamma.lactone**																			
**D.galacturonic.acid**	-0.909***	-0.92***	-0.866***	-0.852***		-0.852***	-0.846***	0.713**	-0.887***	0.851***	-0.873***	-0.911***	-0.845***	-0.931***			0.903***		
**X2.Hydroxy.benzoic.acid**	0.841***	0.768**					0.811***	-0.775**	0.792**	-0.839***	0.797**	0.799**		0.774**			-0.738**	-0.744**	
**X4.Hydroxy.benzoic.acid**																			
**Hydroxybutiric.acid**																			
**Itaconic.acid**																			
**ketobutyric.acid**																			1***
**malic.acid**																			
**arginine**																			
**asparagine**		-0.706**	-0.708**	-0.711**		-0.711**							-0.726**	-0.742**					
**phenylalanine**																			
**serine**																			
**threonine**																			
**glutamic.acid**				0.709**		0.709**							0.741**						
**phenylethylamine**																			
**putrescine**	0.853***	0.822***	0.849***	0.833***		0.833***	0.846***	-0.938***	0.874***	-0.887***	0.887***	0.852***	0.715**	0.715**				-0.935***	

Table.S10 Pearson correlation between the bacterial diversity and the substrate utilization patterns (Biolog Ecoplates). The r-value is provided for significant correlations (**P<0.01 and ***P<0.001).

	Acidobacteria	Bacteroidetes	Chloroflexi	Firmicutes	Gemmatimonadetes	Nitrospira	Alphaproteobacteria	Betaproteobacteria	Deltaproteobacteria	Gammaproteobacteria	Proteobacteria uncl.	Acidimicrobiales	Actinomycetales	Solirubrobacterales	Actinomycetales uncl.	TM7	Verrucomicrobia	Unclassified
**pyruvic.acid.methyl.ester**			-0.562**		-0.634***	-0.611**			-0.611**		-0.535**			-0.548**			-0.542**	
**tween.40**					-0.649***	-0.562**			-0.562**				0.587**				-0.558**	
**tween.80**																		
**alpha.cyclodextrin**		0.607**		0.56**		0.588**		0.631***	0.631***		0.601**	0.555**						
**glycogen**	-0.734***	0.75***	0.79***	0.774***	0.722***	0.833***		0.875***	0.882***		0.881***	0.759***		0.725***			0.584**	
**D.cellubiose**																		
**alpha.D.Lactose**																		
**beta.methyl.D.glucoside**																		
**D.xylose**																		
**i.Erythritol**																		
**D.Mannitol**														-0.525**		0.654***		
**N.Acetyl.D.Glucosamine**																		
**D.Glucosaminidic.acid**																		
**Glucose.1.phosphate**													-0.606**					
**Glycerol.phosphate**			0.582**		0.545**				0.531**									
**D.galactonique.acid.gamma.lactone**																		
**D.galacturonic.acid**																		
**X2.Hydroxy.benzoic.acid**	-0.539**	0.616**	0.568**	0.682***	0.571**	0.661***		0.651***	0.701***		0.642***	0.571**		0.704***				
**X4.Hydroxy.benzoic.acid**																		
**Hydroxybutiric.acid**																		
**Itaconic.acid**																		
**ketobutyric.acid**																0.518**		
**malic.acid**					0.562**													
**arginine**													0.72***			0.58**		
**asparagine**			-0.567**	-0.543**	-0.664***	-0.576**		-0.523**	-0.601**		-0.535**							
**phenylalanine**																		
**serine**																		
**threonine**																0.525**		
**glutamic.acid**																		
**phenylethylamine**		0.52**	0.634***	0.659***	0.604**			0.571**	0.644***		0.599**			0.596**				
**putrescine**	-0.704***	0.77***	0.703***	0.774***		0.7***		0.779***	0.761***		0.787***	0.719***		0.772***	0.648***			
